# Cytokine Storm in COVID-19: The Current Evidence and Treatment Strategies

**DOI:** 10.3389/fimmu.2020.01708

**Published:** 2020-07-10

**Authors:** Yujun Tang, Jiajia Liu, Dingyi Zhang, Zhenghao Xu, Jinjun Ji, Chengping Wen

**Affiliations:** College of Basic Medical Science, Zhejiang Chinese Medical University, Hangzhou, China

**Keywords:** COVID-19, cytokine storm, treatment strategies, immunoregulation, tocilizumab, antimalarial agents

## Abstract

Severe acute respiratory syndrome coronavirus 2 (SARS-Cov-2) is the pathogen that causes coronavirus disease 2019 (COVID-19). As of 25 May 2020, the outbreak of COVID-19 has caused 347,192 deaths around the world. The current evidence showed that severely ill patients tend to have a high concentration of pro-inflammatory cytokines, such as interleukin (IL)-6, compared to those who are moderately ill. The high level of cytokines also indicates a poor prognosis in COVID-19. Besides, excessive infiltration of pro-inflammatory cells, mainly involving macrophages and T-helper 17 cells, has been found in lung tissues of patients with COVID-19 by postmortem examination. Recently, increasing studies indicate that the “cytokine storm” may contribute to the mortality of COVID-19. Here, we summarize the clinical and pathologic features of the cytokine storm in COVID-19. Our review shows that SARS-Cov-2 selectively induces a high level of IL-6 and results in the exhaustion of lymphocytes. The current evidence indicates that tocilizumab, an IL-6 inhibitor, is relatively effective and safe. Besides, corticosteroids, programmed cell death protein (PD)-1/PD-L1 checkpoint inhibition, cytokine-adsorption devices, intravenous immunoglobulin, and antimalarial agents could be potentially useful and reliable approaches to counteract cytokine storm in COVID-19 patients.

## Introduction

In December 2019, an outbreak of a novel coronavirus-based disease was reported in Wuhan, China. On 11 February 2020, the World Health Organization (WHO) named this coronavirus “severe acute respiratory syndrome coronavirus 2” (SARS-CoV-2) and the disease that it caused “coronavirus disease 2019” (COVID-19). As of 25 May 2020, SARS-CoV-2 has affected over 212 countries, and about 5,529,195 cases have been confirmed around the world, of which 347,192 people have died.

The reason for these deaths is suspected to be the “cytokine storm” [also called “cytokine storm syndrome” (CSS)]. The International Classification of Diseases (ICD) does not include the cytokine storm or CSS. Cron and Behrens bring the current knowledge of CSS (1). They define that “cytokine storm” is an activation cascade of auto-amplifying cytokine production due to unregulated host immune response to different triggers. The triggers involved infections, malignancy, rheumatic disorders, etc. Another scholar described that cytokine storm is a systemic inflammatory response to infections and drugs and leads to excessive activation of immune cells and the generation of pro-inflammatory cytokines (2). A similar entity is termed “cytokine release syndrome” (CRS), which is not defined in the textbook of CSS (1). CRS is an acute systemic inflammatory syndrome characterized by multiple-organ dysfunction (MOD). It has been reported that chimeric antigen receptor (CAR)-T-cell therapy could help to distinguish CRS from a cytokine storm (2). Of note, the textbook described the criteria of CSS based on hemophagocytic lymphohistiocytosis (HLH) and secondary HLH (sHLH) associated with rheumatic disorders, such as macrophage activation syndrome (MAS) (1). Thus, it may be not applicable in COVID-19 because the COVID-19 is a contagious disease and relatively irrelevant to a genetic disorder. Up to date, there is still a lack of clinical and laboratory criteria to identify the cytokine storm. In this review, we referred COVID-19 associated cytokine storm as the patients who are severely ill along with a high concentration of pro-inflammatory cytokines.

For patients with COVID-19, the number of white blood cells, neutrophils, as well as levels of procalcitonin, C-reactive protein, and other inflammatory indices, are significantly higher in the intensive care unit (ICU) cases than in non-ICU cases (3, 4). Many studies showed that severely ill patients tended to have a higher concentration of pro-inflammatory cytokines, especially interleukin (IL) 6, than moderately ill patients in COVID-19 (5–9). The result of the bronchoalveolar lavage fluid (BALF) cells, which tested by transcriptome sequencing, reveals excessive chemokines releasing caused by SARS-CoV-2 infection, such as CXCL10 and CCL2 (10). The high level of cytokines also indicates a poor prognosis in COVID-19 (6, 11, 12). Furthermore, the pathology of postmortem examination of the lung, from who was died of COVID-19, demonstrated the existence of acute respiratory distress syndrome (ARDS) and T-cell overactivation (13). This phenomenon is due to an increase in the number of T-helper (Th) 17 cells and the high cytotoxicity of the CD8^+^ T cells (13). The innate and adaptive immune responses activated by SARS- CoV-2 infection lead to uncontrolled inflammatory responses and ultimately cause the cytokine storm (14). The cytokine storm can lead to apoptosis of epithelial cells and endothelial cells, and vascular leakage and, finally, result in ARDS, other severe syndromes, and even death (15).

To lower mortality due to cytokine storm, we summarized the clinical and pathology features of the coronavirus-related cytokine storm. We explored the efficacy and safety of potential treatments and their molecular mechanism. There is still lacking sufficient evidence supporting the regulation of cytokine expression may be beneficial to the mortality of COVID-19.

## What Do We Learn From Other Coronavirus Infections?

The early-stage clinical characteristics of MERS and SARS are influenza-like symptoms (16–18): pyrexia, sore throat, dry cough, myalgia, and dyspnea. Those symptoms are very similar to the characteristics of early COVID-19 and progress rapidly to pneumonia (3, 19, 20). It has been found that the regulation of several cytokines is disordered in the peripheral blood of SARS patients, as summarized by Chen and colleagues (21) and listed in Table 1. Table 1 shows an increase in levels of cytokines and chemokines and a decrease in levels of anti-inflammatory cytokines such as IL-10. Of note, the release of pro-inflammatory cytokines, especially interferon (IFN)-α and IFN-γ, is correlated with lethal SARS (22, 23). The cytokines with increased levels in fatal SARS are IL-6, IL-1β, IFN, and CXCL10. These cytokines are secreted mainly by dendritic cells (DCs) and macrophages, indicating that innate immunity plays a pivotal part in lethal SARS. CCR4^+^ CCR6^+^ Th17 cells have many chemokine receptors and may share the same mechanism and function in cell-cell interactions in SARS. Cytokines secreted by DCs and macrophages induce the infiltration and recruitment of pro-inflammatory Th17 cells. Analyses of lungs from SARS patients have revealed diffuse alveolar damage as a crucial feature. Histopathological studies have shown lung consolidation and edema with pleural effusions and focal hemorrhage, all of which resemble COVID-19 features (13, 24). Besides, the lungs of SARS patients are infiltrated extensively with neutrophils and macrophages, which are not observed in COVID-19. In peripheral blood, numbers of CD4^+^ and CD8^+^ T cells are reduced in cases of COVID-19 and SARS (13, 25) and are associated with death in the latter (25). Interestingly, unlike MERS and SARS, a high concentration of pro-inflammatory CC chemokine receptor (CCR)4^+^ CCR6^+^ Th17 cells are found in COVID-19 (13).

**Table 1:** Cytokine and chemokine responses detected in plasma or serum of SARS patients [adapted from Chen and Subbarao (21)].

**Immune mediator**	**Method of detection (number of patients studied)**						
	**CBA (20)**	**CBA (8 children)**	**CBA/ELISA (88)**	**CBA/qPCR (255)**	**ELISA (288)**	**ELISA (15)**	**LiquiChip (23)**
**Proinflammatory cytokines**							
IL-6	E	–	E	ND	E	–	E
IL-1β	E	E	ND	ND	ND	ND	–
IL-12	E	–	ND	ND	ND	ND	–
TNF-α	–	–	–	ND	–	–	–
**Inflammatory cytokines**							
IFN-γ	E	ND	E	ND	–	ND	–
IL-2	–	ND	–	ND	ND	L	–
IL-4	–	ND	–	ND	–	ND	–
IL-10	–	–	–	ND	L	–	–
IL-13	ND	ND	–	ND	ND	ND	ND
IL-18	ND	ND	E/F	ND	ND	ND	ND
TGF-β	ND	ND	L	ND	–	E	ND
**Chemokines**							
IL-8/CXCL8	E	–	F	E	–	E	E
MIG/CXCL9	–	ND	E/F	E	ND	ND	ND
IP-10/CXCL10	E	ND	E/F	E	ND	ND	E
MCP-1/CCL2	E	ND	E/F	–	ND	ND	E
RANTES/CCL5	–	ND	–	–	ND	ND	–
PGE2	ND	ND	ND	ND	ND	E/L	ND

*CBA, cytometric bead array; qPCR, quantitative polymerase chain reaction; E, elevated in early phase (< 2 weeks); L, elevated in late or convalescent-phase; F, elevated in the fatal case; –, not elevated; ND, not done*.

The innate and adaptive immune system takes multiple measures to respond to virus infection. MERS-CoV infects human epithelial cells and leads to these cells inducing significant but delayed responses by IFN, pro-inflammatory cytokines (e.g., IL-1β, IL-6) and chemokines (e.g., IL-8) (26, 27). SARS-CoV infects airway epithelial cells and results in delayed release of chemokines such as CCL3, CCL5, CCL2, and CXCL10 (28). Besides, MERS-CoV infects hematopoietic cells such as monocytes, macrophages, and DCs, which is not seen in those cells upon SARS-CoV infection (29–32). MERS-CoV infects the cells mentioned above to induce delayed (but increased) levels of pro-inflammatory cytokines (e.g., IL-2) and chemokines (e.g., CCL2, CCL3) (27, 30). Although SARS-CoV is abortive in macrophages and DCs, the virus induces an increase in levels of pro-inflammatory cytokines and chemokines (31, 32). SARS-CoV and SARS-CoV-2 infect cells using the same receptor: angiotensin-converting enzyme-2 (33). Hence, it has been postulated that both viruses can affect the same spectrum of cells.

In the aspects of murine models of coronavirus, infection with SARS-CoV in BALB/c mice has been shown to induce an increase in the number of pathogenic inflammatory monocyte–macrophages (IMMs) (34). Through stimulation of IFN-α/β receptors, the accumulating IMMs produce monocyte chemokines (e.g., CCL2, CCL7, CCL12) and pro-inflammatory cytokines [e.g., tumor necrosis factor (TNF), IL-6, IL1-β], which results in further accumulation of pathogenic IMMs. Targeting of IFN signaling, IMMs, or pro-inflammatory cytokines could offer protection from lethal SARS-CoV infection. In this way, the chemokines (produced by activated monocytes and macrophages) lead to the recruitment of neutrophils, monocytes, and T cells into the lungs (28). After chemotaxis, activated effector T cells migrate to the lungs and destroy pneumocytes/permissive cells due to response to the virus infection (35). The damage caused by neutrophils, monocytes, and T cells results in lung-parenchyma changes, such as diffuse alveolar damage, which leads to ARDS (35).

In summary, the excessive cytokines and chemokines caused by lethal coronavirus infection involve mainly antigen-presenting cells (APCs) (such as macrophages) and T cells. However, cytokines secreted by immune cells are produced to eliminate viral infection, and deficiency of such cytokines may be harmful to the body. For example, virus titers are significantly higher in toll-like receptor (TLR)3^−/−^, TIR-domain-containing adapter-inducing interferon-β (TRIF)^−/−^, and IL-6^−/−^ mice compared with their wild-type counterparts, and are associated with severe lung damage (36, 37).

## Clinical, Immunological and Pathologic Features of COVID-19 Associated Cytokine Storm

In China, we classified the stage of COVID-19 according to the guidelines (38) issued by the National Health Commission of the People's Republic of China (NHC). According to the instructions, NHC defines severe illness of COVID-19 as one of the following conditions: respiratory rate ≥30 breaths/min in the resting state; Oxygen saturation ≤93%; arterial blood oxygen partial pressure (PaO_2_)/fraction of inspired oxygen concentration (FiO_2_) ≤300 mmHg. Critical illness as one of the following conditions: respiratory failure and requiring mechanical ventilation; shock; complication of other organ failures, and needs intensive care. The most common symptoms of COVID-19 were fever, cough, shortness of breath, fatigue, and myalgia (5, 7, 39, 40), and severe cases tend to be older with more basic diseases and suffer from dyspnea, more complications (5, 40). In COVID-19, 14% of patients progress to severe disease and 5% to critical illness (41). A prospective study reported that the computerized tomography (CT) of the lungs of COVID-19 (6). The lung lesions increase and the scope expands as the disease progresses, and ground-glass opacity coexisted with consolidation or striated shadow. Some severe patients showed diffuse lesions in both lungs.

Up to date, the inflammatory disorders (insufficient in chemokines) in COVID-19 have been reported in many clinical studies. The COVID-19 is inclined to cause a decrease of lymphocyte count and an increase of C reactive protein (CRP), especially in severely ill patients (5–7, 42–44). The major subsets of the T lymphocytes (T cell) (CD3^+^ CD4^+^ T cell and CD3^+^ CD8^+^ T cells) are reduced in the COVID-19 and are significantly lower in the severe cases (5, 12, 42, 43, 45, 46); however, controversial results are also reported in some studies (7, 40). The results of the other immune cells, the B cell and natural killer (NK) cell, have more inconsistency in recent researches. IL-6 was observed increased in all studies, and only one study show IL-10 was not elevated. About half of the studies we collected showed TNF-α was increased. Only Huang et al. (9) inspected the multiple types of chemokines and found that severe patients had higher levels of G-CSF, GM-CSF, IP-10, MCP-1, MIP-1a, MIP-1b, RANTES, and IL-8. The inflammatory disorders of COVID-19 were summarized in Table 2.

**Table 2:** Cytokine, chemokine, and leukomonocyte responses detected in COVID-19 patients.

**Comparison objects**	**IL-6**	**IL-1β**	**IL-10**	**TNF-α**	**IFN-γ**	**IL-2(R)**	**IL-4**	**IL-8**	**IL-17**	**CD3^**+**^ CD4^**+**^ T cell**	**CD3^**+**^ CD8^**+**^ T cell**	**B cell**	**NK cell**	**References**
SC (*n* = 11) vs. MC (*n* = 10)	I	ND	I	I	D	I	ND	ND	ND	D	D	ND	ND	(5)
SC (*n* = 9) & CC (*n* = 5) vs. MC (*n* = 15)	I	–	–	–	ND	I	ND	–	ND	ND	ND	ND	ND	(6)
SC (n−27) vs. MC (*n* = 17)	I	–	I	I	ND	ND	ND	I	ND	D	–	–	–	(7)
ICU care (*n* = 13) vs. No ICU care (*n* = 28)	I	–	I	I	–	ND	I	I	–	ND	ND	ND	ND	(9)
SC (*n* = 37) & CC (*n* = 16) vs. MC (*n* = 57)	I	–	I	ND	ND	ND	ND	–	ND	D	D	ND	ND	(12)
SpO_2_ < 90% (n = 7) vs. SpO_2_ ≥90% (*n* = 36)	I	ND	I	–	ND	–	–	ND	ND	–	–	–	ND	(40)
SC (*n* = 34) vs. MC (*n* = 67)	ND	ND	ND	ND	ND	ND	ND	ND	ND	D	D	D	D	(42)
SC (*n* = 30) vs. MC (*n* = 125)	I	ND	ND	ND	ND	ND	ND	ND	ND	D	D	ND	ND	(43)
SC (*n* = 269) vs. MC (*n* = 279)	I	–	I	I	ND	I	ND	–	ND	ND	ND	ND	ND	(44)
SC (*n* = 21) vs. MC (*n* = 102)	I	ND	I	–	–	–	–	ND	–	D	D	–	–	(45)
SC (*n* = 45) & CC (*n* = 62) vs. MC (*n* = 80)	I	–	I	ND	ND	ND	ND	ND	ND	D	D	D	D	(46)

*SC, severe cases; CC, critical cases; MC, moderate cases; I, increased; D, decreased; –, not elevated; ND, not done*.

The pathologic features of COVID-19 showed the lungs were infiltrated with excessive CCR6^+^ Th17 cells and high cytotoxicity of CD8^+^ T cells (13). But high cytotoxicity of CD8^+^ T cells does not mean they exert the normal function. The SARS-CoV-2 could lead to cytotoxic lymphocytes (mainly involving NK cells and CD8^+^ T cells) exhaustion, which is manifested as the upregulated exhaustion markers, such as NKG2. The exhaustion markers return to normal in patients who have recovered or are convalescent (47, 48). BALF cells were found extreme cytokine releases, such as CCL2, CXCL10, CCL3, and CCL4 (10). Furthermore, Xiong et al. (10) use the transcriptome dataset approach to discover that SARS-CoV-2 can activate apoptosis and P53 signaling pathway (one of the pathways responsible for the survival of the cell) in lymphocytes. These results could provide some reasons for the cause of patients' lymphopenia. Another team of Chen and his colleagues studied the mechanisms for lymphopenia (49). Their results demonstrate that SARS-CoV-2 infected the CD169^+^ macrophages in spleens and lymph nodes (LNs), and lead to lymphoid tissue damage, such as splenic nodule atrophy and lymph follicle depletion, etc. The CD169^+^ macrophages express high Fas and cause activation-induced cell death (AICD) through Fas/FasL interactions. Furthermore, SARS-CoV-2 selectively induced macrophages to produce IL-6, not TNF-α and IL-1β, to directly promotes lymphocyte necrosis. The analysis of peripheral blood mononuclear cells (PBMCs) revealed that non-structural protein (nsp) 9 and nsp10 of SARS-CoV-2 target NKRF (NF-κB repressor) to promote IL-6/IL-8 production (50). As a consequence, it recruits neutrophils and induces uncontrollable host inflammatory response.

Collectively, the clinical, immunological, and pathologic features of COVID-19 have something in common with SARS and MERS. For example, all the viruses can cause lymphopenia and influenza-like symptoms in the early stage. SARS and COVID-19 do not lead to the upgrade of TNF-α, but the increase of IL-6 and IL-10 is more prevalent in COVID-19. The IL-6 plays a crucial role in the pathologic of COVID-19, including the chemotaxis of neutrophils and lymphocyte necrosis. Importantly, COVID-19 is more able to cause cytotoxic lymphocytes exhaustion.

## Potential Treatments for Cytokine Storm in COVID-19 and Their Safety

### IL-6 Inhibition

Tocilizumab (TCZ) is a recombinant humanized anti-human IL-6 receptor monoclonal antibody, preventing IL-6 binding to its receptor to exert the immunosuppression promoted by IL-6. Michot et al. (51) reported that 42-year-old male suffering from respiratory failure due to SARS-CoV-2 infection. After 4 days of TCZ treatment, the CRP decreased from 225 to 33 mg/L and ultimately clinically fully recovered. Similarly, some case reports showed TCZ is an efficacy and safety approach in COVID-19, even patients with other diseases combined, such as multiple myeloma, end-stage renal disease, and sickle cell disease (52–54). Recently, a retrospective study (55) found that TCZ decreased CRP in all patients (*n* = 15) rapidly, but three of them, who are critically ill, still dead. The dead patients show continuously rising of IL-6 even after the administration of TCZ and methylprednisolone, indicating that repeat doses of TCZ may be needed in COVID-19 patients who are critically ill. Another retrospective study (56) demonstrated that TCZ showed a quick control of severe COVID-19 manifestation, such as fever, respiratory function. All patients (*n* = 21, two were critically ill), have recovered and have been discharged from hospital, and no adverse event was reported during the treatment. A prospective open-label, multicenter single-arm study manifests the pilot results of the off-label application of TCZ in severe patients with COVID-19 (57). The study involved 63 patients with severe COVID-19, and TCZ succeeded in improving respiratory and laboratory parameters, such as Pa0_2_, Fi0_2_, consequently, increased the likelihood of survival (the death rate of the study is 11%). It is worth mentioning that a cautionary case report by Radbel et al. (58). Two patients were diagnosed with COVID-19 complicated by CRS and treated with TCZ. Unfortunately, both patients progressed to severe HLH, and one developed to viral myocarditis.

All the cytokines produced by immune cells are responsible for viral clearance. Suppression of cytokine release at an early stage of disease as treatment is controversial. Application of synthetic disease-modifying antirheumatic drugs (DMARDs) and biologic DMARDs to downregulate cytokine expression in RA increases the risk of infection (59, 60). The timing and the doses of the intervention still need to be inspected clearly. SARS-CoV-2 mainly causes a dramatic increase in IL-6 and does not remarkably promote other pro-inflammatory factors, such as IL-1β and IFN-γ. Although treating COVID-19 with TCZ is an off-label use, it may be relatively appropriate and safe in coping with COVID-19 associated cytokine storm basing on the current evidence. It still needs more large samples and high-quality studies to evaluate the exact efficacy and safety in COVID-19. The ongoing trials of potential treatments and other treatments focus on inflammatory disorders in COVID-19 are available in Supplementary Table 1.

### Corticosteroids

Glucocorticoid therapy is used widely among critically ill patients with other coronavirus infections (e.g., SARS, MERS). Corticosteroids have been administered to ICU patients infected with SARS-CoV-2 (3, 4, 20). Glucocorticoids exhibit pharmacologic effects at any therapeutically relevant dose through classic genomic mechanisms. Some immunosuppressive effects are based on transactivation, and glucocorticoid induces gene transcription and protein synthesis of NF-κB inhibitors and lipocortin-1. Through inhibition of NF-κB signaling, glucocorticoids induce inhibition of synthesis of downstream proteins such as IL-1, IL-6, granulocyte-macrophage colony-stimulating factor, and inducible cyclooxygenase-2 (61, 62). Glucocorticoids reduce the proliferation, activation, differentiation, and survival of T cells and macrophages (63). Glucocorticoids proffer inhibitory actions on the transcription and action of various cytokines. The Th1 and macrophage-based pro-inflammatory cytokines IL-1β, IL-2, IL-6, TNF-α, and IL-17 are inhibited by glucocorticoids (63).

However, it is controversial whether corticosteroids are beneficial in the treatment of severe COVID-19 patients. A comment and a meta-analysis, which mainly bases on the evidence of SARS and MERS (64, 65), stated that corticosteroid would increase mortality and delayed clearance of viral in coronavirus infection diseases. Thus, the corticosteroids should not be administrated for the treatment of SARS-Cov-2 induced lung injury or shock. Newly published studies also indicate that the use of corticosteroids is not beneficial for COVID-19 patients (not severe cases), and high-dose corticosteroids are associated with mortality (44, 66, 67). Most COVID-19 patients discussed in these studies are not severe cases. Inspecting the studies included and analyzed by the meta-analysis, only one study (68) described the numbers of patients with corticosteroids and non-corticosteroids treatment in the severe group and non-severe group. The study demonstrated the benefit of corticosteroids use in severe SARS-Cov infection. Another comment (69), which was written by front-line physicians from China, showed corticosteroids might have some benefit for critically ill patients with COVID-19. Systematic corticosteroid therapy could promote oxygen saturation and PaO_2_/FiO_2_. However, corticosteroids might not improve mortality in critical COVID-19 patients.

Current evidence shows that SARS-Cov-2 induces an increase in a small range of cytokines. It might be overuse to administrate corticosteroids to counteract a wide range of cytokines. Furthermore, SARS-Cov-2 causes relatively serious lymphocytopenia and lymphocytes exhaustion. Glucocorticoid-mediated stimulation of the “hypothalamic-pituitary-adrenal axis” might also exacerbate lymphocytopenia (70). Thus, the use of corticosteroid is a double-edged sword in COVID-19. The dose, duration, and timing of corticosteroid therapy will be crucial if administrated to COVID-19 patients.

### PD-1 Checkpoint-Inhibitor

As stated above, lymphocytes exhaustion is one of the characteristics of COVID-19, and PD-1 checkpoint-inhibitor might some help in reversing the anergy of lymphocytes. Up to 4 May 2020, no study of PD-1 checkpoint-inhibitor has been reported in the Treatment of COVID-19. The pathway consisting of the receptor PD-1 and its ligands, PD-L1 and PD-L2, play crucial parts in the maintenance of peripheral tolerance. Treatments with antibodies targeting PD-1/PD-1 ligands have elicited an increased response in different cancer types and, in tandem with antibodies targeting cytotoxic-T-lymphocyte-associated antigen-4, have changed cancer therapy radically (71). Unfortunately, signaling regulated by the PD-1/PD-L pathway is also related to substantial inflammatory effects (e.g., sepsis), as this pathway plays a role in balancing protective immunity and immunopathology (72). Increased PD-L1 expression in monocytes is associated with mortality in patients with septic shock (73). A meta-analysis of checkpoint inhibitors showed that such therapy increased the chance of survival (74). Nivolumab (anti-PD-1) and BMS-936559 (anti-PD-L1) had completed phase-Ib randomized studies for severe sepsis. They revealed that giving a checkpoint inhibitor did not result in unexpected safety findings or indicate a cytokine storm (75, 76). Also, CD4^+^ and CD8^+^ T cells were hyperactivated, as revealed by the high proportions of human leukocyte antigen-DR isotype and CD38, in COVID-19; CD8^+^ T cells harbored high levels of cytotoxic granules in COVID-19 patients, in which the phenotype is similar to fatal H7N9 disease (13, 77). Those results suggest that lethal COVID, along with H7N9, may be related to defective activation and exhaustion of T cells, which also suggest that checkpoint-inhibitor administration may reverse this status.

### Cytokine-Adsorption Device

Cytokine adsorption involves using a method, such as extracorporeal membrane oxygenation (ECMO), to filter harmful substances directly. An extracorporeal cytokine hemoadsorption device called Cytosorb® (Cytosorbents, Monmouth, NJ, USA) has been reported to capture and reduce inflammatory mediators. Bruenger and colleagues reported that the plasma level of IL-6 and procalcitonin decreased in one patient with severe ARDS after Treatment with ECMO using a hemoadsorption device (78). A 45-year-old patient with severe ARDS showed that venous arterial-ECMO combined with hemoadsorption therapy decreased plasma concentrations of IL-6 and IL-8. Moreover, hemodynamic stabilization, respiratory improvement, and a decline in capillary leakage can be achieved in combination therapy (79). Two trials employing hemoadsorption therapy for infection-related cytokine storm are ongoing (NCT04195126, NCT03685383).

A similar therapy involves dialysis. The mainly water-soluble mediators are removed from plasma, and the hemofilters can have additional adsorptive properties (80). Continuous venovenous hemofiltration and adsorption for severe septic shock are being tested in one clinical trial (NCT03974386).

Neutralizing excessive cytokines with hemoadsorption devices might be relatively effective. The disadvantage is like corticosteroids: a wide range of cytokines would be adsorbed. Thus, it would lead to the a lack of cytokines, which are at reasonable or even insufficient levels. We suggest treating the cytokine storm in COVID-19 should base on the laboratory results of cytokines and chemokines. Meanwhile, adjusting the parameters of the devices (e.g., treatment duration) for preventing overtreatment.

### Intravenous Immunoglobulin (IVIG)

IVIG can elicit passive immunity, anti-inflammatory, and immunomodulatory effects that can improve treatment effects and increase survival in severe infection. An IgG molecule binds to a specific target antigen through the humoral and cellular arms of the immune system. For example, IgG molecule blocks the cell-cell interactions mediated by cell-surface receptors (such as CD95 and CD95 ligand), neutralize the autoantibodies by anti-idiotypic antibodies, expanse the regulatory T (Treg) cell populations via the blockade of immune complex binding to low-affinity Fcγ receptors (FcγRs), to exert the functions of immunomodulation (81). Ma and colleagues detailed a severe case of glandular fever treated with IVIG (82). Levels of Th1 cytokines (IFN-γ, IL-12, soluble tumor necrosis factor receptor 1 (sTNFR1), CXCL10, CXCL9, CCL3), and viral loads eventually recovered after the combination of prednisolone with IVIG. A multicenter, double-blind, randomized controlled trial for cases with severe influenza A (H1N1) infection demonstrated that IVIG reduced the serum concentration of cytokines, viral load, and reduced mortality (83). A meta-analysis of 17 studies (1,958 participants) found IgM-enriched polyclonal and standard Ig molecules decreased mortality in adults with severe sepsis or septic shock. However, a meta-analysis did not reveal a benefit in adult mortality with polyclonal IVIG using high-quality trials only (84).

### Hydroxychloroquine (HCQ)

Despite a lack of clinical evidence, the US gave emergency approval to HCQ, a member of antimalarial agents, in COVID-19 on 28 March (85). A meta-analysis included the studies up to 5 April 2020 (86) and showed that four clinical trials and three observational studies are eligible for the study. Unfortunately, the authors concluded that HCQ has no clinical effect on patients with COVID-19. However, a randomized clinical trial published on 24 April, which included the patients (*n* = 81) with critically ill COVID-19 (such as high respiratory rate, peripheral oxygen saturation lower than 90%, shock), indicated 15.0% patients (6 of 40) have died in the low-dosage group (i.e., 450 mg twice daily on day 1 and once daily for 4 days). The critically ill death rate is over 50%, as reported by WHO (87). Thus, low-dosage of HCQ could be beneficial for critically ill patients with COVID-19. The study also indicates high dosage HCQ might not be suitable for critically ill patients because of its potential safety hazards.

### Other Potential Strategies: Lessons From Chinese Experiences

Traditional Chinese medicine (TCM) has an essential role in the latest SARS epidemic. Several studies (88–93) have shown that the add-on of TCM to Western medicine can shorten the duration of hospitalization, alleviate symptoms, reduce mortality (including for critically ill patients), and reduce the prevalence of adverse reactions in SARS. Compared with a control group (Western medicine only), a combination of TCM with Western medicine has shown advantages in terms of symptom alleviation and preventing COVID-19 (94–96). However, the quality of the studies must be improved. The administration of TCM in a standard manner worldwide is complicated because of the different decoctions used and the matching of herbs.

Artemisinin can be obtained from *Artemisia annua*, and one kind of antimalarial agents. Hou and colleagues showed that extracts from artemisinin-family drugs could regulate cells from the innate and adaptive immune system, and lead to anti-inflammatory and immunomodulatory actions (97). The scope of application for artemisinin-family medicines includes infectious disease and autoimmune diseases, and artemisinin-family shows a difference in immune regulation compared with hydroxychloroquine (98–100).

#### Artemisinin-Family Drugs Ameliorate Infection-Induced Acute Injuries and Reduce Mortality

As stated above, ALI and AKI are crucial mortality factors in infectious diseases. Artesunate is a derivative of artemisinin and can lessen the pathologic changes and neutrophil infiltration in the lungs of ALI patients, and decrease sepsis-induced mortality (101). By inhibiting expression of NF-κB signaling and enhancing heme oxygense-1 expression, the artesunate can lower the concentrations of TNF-α and IL-6 in serum and bronchoalveolar lavage fluid. Huang and colleagues discovered that dihydroartemisinin could attenuate lipopolysaccharide (LPS)-induced ALI through suppressing NF-κB signaling in a nuclear factor erythroid 2-related factor 2 (Nrf2)-dependent fashion, thereby leading to a decrease in expression of the pro-inflammatory cytokines IL-1β, TNF-α, and IL-6 (102). Hu and colleagues explored a new and efficacious approach for ALI (103). “Artesunate liposomes” were prepared using film dispersion and then lyophilized to obtain liposomal artesunate dry powder inhalers (LADPIs). After treatment with LADPIs, a rapid reduction in accelerated inhalation, ALI syndromes, and levels of TNF-α and IL-6 has been observed in rats. Besides, kidney impairment in hospitalized COVID-19 patients is associated with a high risk of in-hospital death (104). Cheng et al. (105) observed that dihydroartemisinin lessened glomerular injury and relieving increases in the urine albumin: creatinine ratio and serum levels of creatinine.

#### Artemisinin-Family Drugs Regulate Immune Cells and Their Molecular Mechanisms

Current evidence of pathologic changes of COVID-19 suggests the dysregulation of the cytokines involves mainly macrophages/monocytes. In a burn-based sepsis model BALB/c mice, concentrations of adhesion molecules and neutrophil infiltration in the lungs and heart, and mortality rate are significantly increased, but those phenotypes could be reversed by artemisinin (106). The authors discovered that artemisinin downregulates protein levels of NOD-, LRR- and pyrin domain-containing protein 3 (NLRP3) and caspase 1 in macrophages in burn-induced sepsis mice. Also, a reduction in levels of the pro-inflammatory cytokines IL-1β and IL-18 has been observed post-therapy. NLRP3 is a sensor component expressed mainly in macrophages and which undergoes transcription by NF-κB. NLRP3 is responsible for the maturation and secretion of IL-1β and IL-18 (107–109). NF-κB also increases the level of IL-10 in the macrophages infected by *Plasmodium falciparum*, and artemisinin could reduce IL-10 production in animal models (110), as well as in the clinic (111).

Two studies focused on the relationship among TLR, NF-κB, nucleotide-binding oligomerization domain-containing protein (NOD)2, and macrophages. TLR2 mainly locates outside the cell membrane of macrophages, DCs, and granulocytes, and recognizes bacteria (112). TLR2 induces NF-kB activation through recruitment of TIR Domain Containing Adaptor Protein (TIRAP) and myeloid differentiation primary response (MyD)88 in macrophages and DCs. In inflammatory monocytes, TLR2 is expressed within endosomes and induces the release of type-I IFNs *via* Interferon regulatory factor 3 (IRF3) and IRF7 in response to viruses (113). Artesunate increases survival of mice challenged with live *Staphylococcus aureus*/methicillin-resistant *Staphylococcus aureus* (MRSA) compared with antibiotics alone, and its protection may be associated with reductions in TNF-α levels. Artesunate reduces the expression of TLR2 mRNA and Nod2 mRNA that upregulated by *S. aureus*/MRSA and also inhibits the activation of NF-κB (114). Kuang and colleagues found that the artesunate attenuated the release of TNF-α and IL-6 from macrophages by inhibiting TLR4-mediated autophagic activation (115). TLR4 also locates in the endolysosomal compartment, can recognize Gram-negative bacteria and viruses (112), shares the same pathway as the activation of NF-κB, and induces the release of type-I IFNs *via* the TNF receptor-associated factor (TRAF3)- TANK Binding Kinase 1 (TBK1)-IRF3 axis (113). However, Kuang and co-workers discovered that artesunate attenuates the cytokine release by the TRAF6-beclin1- Class III phosphatidylinositol 3-kinase (PI3KC3) pathway. In a model of severe acute pancreatitis in rats, artesunate attenuates the release of IL-1β and IL-6 *via* the TLR4-NF-κB axis (116). In addition, dihydroartemisinin inhibited the activation of TLR4 and IRF3 in the spleen cells of systemic lupus erythematosus (SLE)-prone MRL/lpr mice, which lead to a decrease in levels of IFN-α and IFN-β (117).

The mitogen-activated protein kinase (MAPK) signaling pathway plays a vital part in the development, differentiation, proliferation, transformation, and apoptosis of cells (118). The extracellular signal-regulated kinase (ERK), JNK/Stress-activated protein kinases (SAPK), and p38 MAPK are the dominant members of the MAPK family. The cascades can be summarized as the ERK pathway (Raf-MEK-ERK), JNK pathway (TAK1-MKK-JNK), and p38 pathway (TAK1-MKK-p38). Pro-inflammatory cytokines such as IL-1 and TNF-α, IFNα, and IFNγ can induce activation of the p38 pathway, and p38 can regulate NF-κB-dependent transcription after its nuclear translocation. Meanwhile, NF-κB is a crucial transcriptor for IL-6, which could activate the IL-6-janus kinase (JAK)-signal transducer and activator of transcription (STAT) pathways (119). Wang and colleagues (120) found that another artemisinin derivative, SM905, suppressed generation of nitric oxide, TNF-α, IL-1β, and IL-6 in LPS-induced macrophages. The underlying mechanism was that SM905 reduced activation of p38 and ERK, and JNK suppressed IκBα degradation. Furthermore, they observed that NF-κB was inhibited correspondingly in SM905-treated cells. In another LPS-induced macrophage model, artemisinin has a property of prohibiting STAT1 activation, and it leads to the reduction of NO (an inflammatory-cascade inducer) in macrophages (121). Except for STAT1, STAT3, and STAT5 in the splenocytes of SLE-prone MRL/lpr mice could be inhibited by SM934, an artemisinin derivative (122).

Artesunate therapy has been shown to improve the survival of mice infected with the herpes simplex virus. Artesunate can lower levels of IL-1β, IL-2, IL-6, IFN-γ, CCL2, CCL3, and CCL4 in these mice. These cytokines are produced primarily by APCs and Th1 cells. Previous studies have suggested that the artesunate can regulate Th cells in virus infections. Du and colleagues (123) demonstrated that the artesunate downregulated the Th1 response and reduced levels of IFN-γ, TNF-α, IL-12, IL-18, CCL2, CXCL9, and CXCL10 in an experimental model of cerebral malaria. RA is an autoimmune disease manifested by dysfunction of various immune cells (e.g., APCs, Th1, Th17), which leads to a high concentration of IL-1, IL-6, TNF-α, and chemokines in plasma and tissues (124). In the experimental models of RA, the proliferation of Th17 cells and the production of IL-17A and IL-6 are inhibited by SM905 therapy and, correspondingly, the expression of retinoic acid receptor-related orphan nuclear receptor gamma t (RORγt) (a specific transcription factor for Th17 cells) is also reduced (125). Fan et al. (126) demonstrated similar data and found that DC32 (an artemisinin derivative) can restore the T_reg_/Th17 balance and reduce transcription of CXCL12 and CX3CL1. T_reg_ can be anti-inflammatory, secrete anti-inflammatory cytokines (e.g., IL-10), target Th17 cells and macrophages, as well as reduce the concentration of IL-1, IL-6, TNF-α, and IL-17 (127). The immunosuppressive mechanisms of artemisinin on T cells include inhibiting differentiation of Th17 cells by regulating the expression of RORγt and maybe also inhibition of activation of the ERK pathway (Ras-Raf1-ERK1/2) (128). In the model of RA-fibroblast-like synoviocytes (FLS), artesunate decreased the production of IL-6, IL-8, and IL-1β through preventing NF-κB translocation and IκBα degradation (129).

#### Artemisinin-Family Drugs in a Clinical Study

Artemisinin-family drugs have shown efficacy and safety in treating malaria. One study reported 32 patients with severe malaria caused by *Plasmodium falciparum*. Ten patients suffered renal failure, eight had cerebral malaria, and 14 had other causes of severe malaria. After artesunate treatment, concentrations of IL-6, and soluble IL-6 receptor in plasma were normalized within 24 h (130).

In recent years, artemisinin-family drugs have been shown to be beneficial against infection caused by the human cytomegalovirus, hepatitis-B virus, Ebola virus, and human immunodeficiency virus (131). Shapira and co-workers reported the first case of the Treatment of HCMV infection with artesunate (132). Germi and collaborators (133) reported that the artesunate led to an effective response in three cases with mild HCMV infection but was not efficacious in two patients with severe HCMV infection.

## Discussion

The elevations of IL-6 and IL-10 are highly consistent in COVID-19. IL-6 targets the IL-6 receptor, and the letter recruit JAK, which transit cascade signal to activate signal transducer and activator of transcription 3 (STAT3) (119). Some physicians suggest tofacitinib, a small molecule compound target JAK1 and JAK3, could be applied in the treatment of COVID-19, and tofacitinib success in treating a COVID-19 patient complicated with ulcerative colitis (134). IL-10, a cytokine with anti-inflammatory properties, could be secreted by virtually all immune cells, including macrophages, DCs, NK cells, T cells, and B cells (135). We might tend to regard the high levels of IL-10 as negative feedback of counteracting the increase of IL-6 because IL-10 can block the activity of NF-κB to downregulate the production of IL-6 (135). However, an abundance of IL-10 also inhibits the function and proliferation of immune cells (e.g., Th1, NK cells, and CD8 T cells), which delays the clearance of viruses (135). Therefore, a mass of IL-10 might be responsible for the normal levels (one study report low level) of IFN-γ (a cytokine for the clearance of viruses) and the exhaustion of lymphocytes. The IL-10 inhibitor in the treatment of COVID-19 also needs to be considered. Even the combination of IL-10 and IL-6 inhibitor could be designed in future prospective studies. When using any method to regulate the dysregulation of cytokines, we might better closely monitor the laboratory index for preventing over-treatment. For example, if we use TCZ to reduce the levels of IL-6, we could check IL-6 levels once every 2 days to keep it at a suitable concentration, which should be studied in the future. Also, the dose and duration would be illuminated.

The current evidence indicates that TCZ, an IL-6 inhibitor, is relatively effective and safe. Based on the therapeutic mechanisms, we classified the remaining therapies, corticosteroids, PD-1/PD-L1 checkpoint inhibition, cytokine-adsorption devices, intravenous immunoglobulin, and antimalarial agents, as “less potential treatments.” No literature of COVID-19 except for corticosteroids mentions the effectiveness and safety of the less potential treatments. The benefits, dose, duration, and timing of corticosteroids still in debate, and the other less potential treatments need clinical evidence to validate.

**Figure 1:**
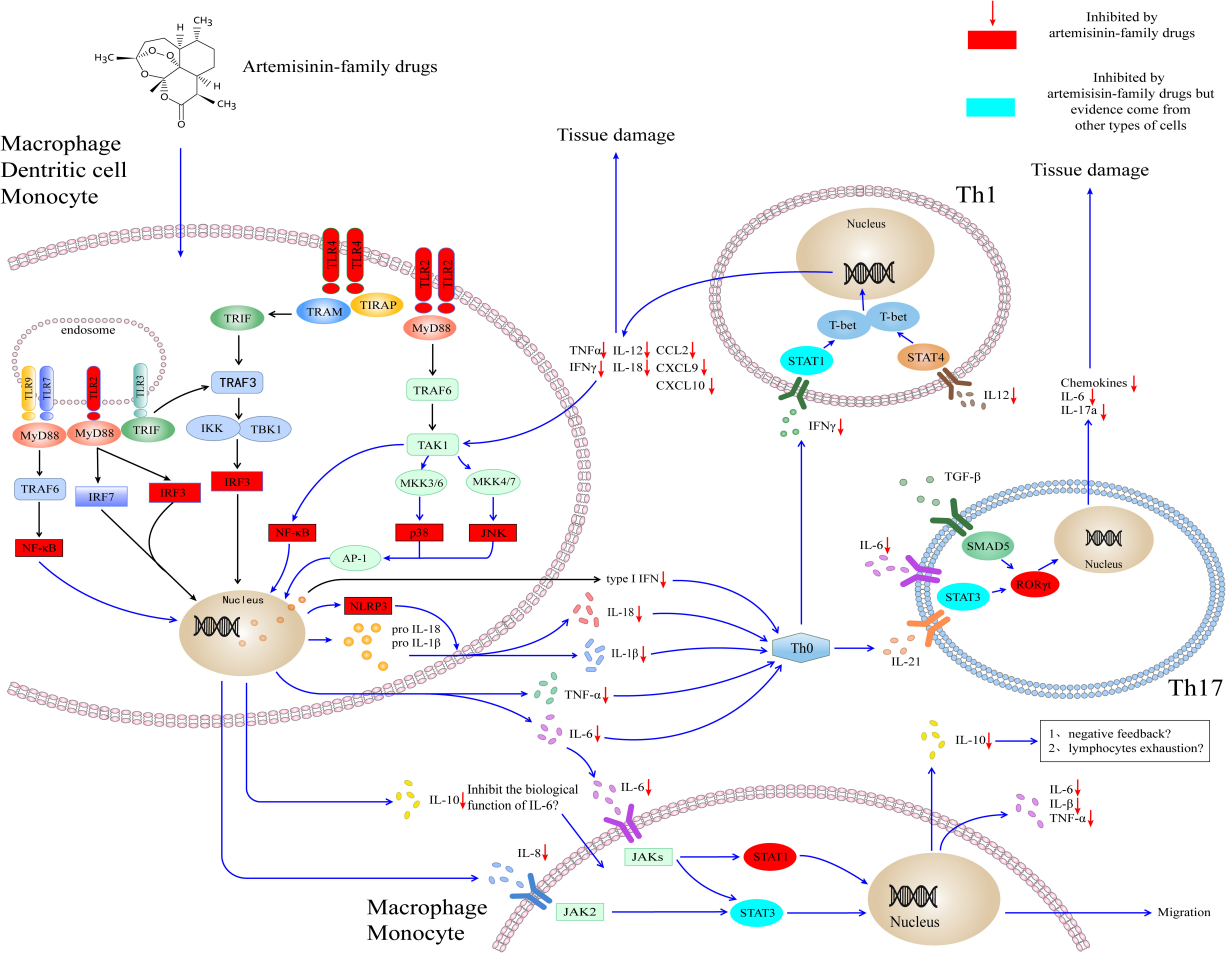
Artemisinin-family drugs for cytokine storm in COVID-19. The dysregulation of the cytokine storm involves mainly APCs. TLR2 and TLR4 locate mainly outside macrophages, DCs, and granulocytes. Also, they are expressed within endosomes, play a role in recognizing bacteria and viruses. Through MyD88-dependent or TRIF-dependent pathway, TLR2 and TLR4 transmit signals for the activation of IRF3 and NF-κB to induce the type I interferon and cytokines. Besides, TLR2 leads to the activation of AP-1, which is responsible for the transcription of inflammatory cytokines. The cytokines target at the naïve T helper cell, to result in the naïve T helper cell to differentiate to Th1 cell and Th17 cell, subsequently to secrete the inflammatory cytokines and chemokines. Moreover, the IL-6, IL-8, and IL-10 secreted by monocytes and macrophages could activate cytokines receptors (i.e., IL-6R, IL-8R), lead to the activation of JAK-STAT signaling pathways and cell migration. The artemisinin-family drugs target at a variety of molecules (red and blueness nodes) in the inflammatory networks, such as NF-κB, IRF3, ERK (not shown in the figure), and RORγt, which inhibit the differentiation of inflammatory cells and the production of cytokines and chemokines. IL-10 is an anti-inflammatory cytokine. It could be secreted by virtually all immune cells, including macrophages, DCs, NK cells, T cells, and B cells. At the moment, the high concentration of IL-10 in severely ill patients with COVID-19 is a mystery. On the one hand, it might play a role in antagonizing the biological function induced by IL-6. On the other hand, the high concentration of IL-10 might contribute to the lymphocytes exhaustion. AP-1, activating protein-1; CCL, C-C motif chemokine ligand; CXCL, C-X-C motif chemokine ligand; IKK, IκB kinase; IFN, interferon; IRF3, interferon response factor 3; JAK, Janus kinase; JNK, Jun N-terminal kinase; MyD88, myeloid differentiation primary response protein 88; NF-κB, Nuclear factor κ B; NLPR3, NOD-, LRR- and pyrin domain-containing protein 3; MKK, Mitogen-activated protein kinase kinase; SMAD5, SMAD Family Member 5; RORγt, retinoic acid receptor-related orphan nuclear receptor gamma t; STAT, Signal transducer and activator of transcription; TAK1, TGFβ-activated kinase; T-bet, T-box transcription factor 21 (also known as TBX21); TLR, Toll-like receptor; TRAF, TNF receptor-associated factor; TRAM, TRIF-related adaptor molecule; TRIF, TIR domain–containing adaptor protein inducing interferon-β. IL, interleukin.

Although the experimental model of infectious disease (e.g., malaria and sepsis) and autoimmune disease (e.g., RA and SLE) indicates that artemisinin-family drugs could target the inflammatory networks to decrease the levels of cytokines (e.g., IL-6 and TNF-α) and chemokines (e.g., IL-8, CXCL10) (Figure 1). The effect and safety of antimalarial agents still need to be validated in the high-quality clinical studies and the SARS-Cov-2 infection disease model.

A precise definition of a cytokine storm is needed urgently. Mehta et al. (136) suggest that the criteria of sHLH could be applied. Moreover, the term needs to be placed in the ICD code. The ICD code would bring the standardization of disease names, the convenience of electronic medical records (EMR) management, and the efficiency in information sharing. For example, the characteristic of cytokine storm would be more accessible to be collected for a retrospective study.

## Author Contributions

YT: manuscript preparation and wrote the main part of the manuscript. JL: evidence collection, wrote the parts of the manuscript, and manuscript editing. DZ: helped to perform the analysis with constructive discussions. ZX: helped to revise the manuscript and gave many professional suggestions. JJ: ideas, formulation of overarching research goals, and aims. CW: critically reviewed the manuscript, project funding, and study initiation. All authors approved the final version of the manuscript.

## Conflict of Interest

The authors declare that the research was conducted in the absence of any commercial or financial relationships that could be construed as a potential conflict of interest.

## Supplementary Material

Supplementary Table 1 Clinical trial registration for inflammatory disorder with COVID-19.
