# Supplementary material for: Cytokine Storm in COVID-19: The Current Evidence and Treatment Strategies
Source: Front Immunol. 2020 Jul 10;11:1708. doi: 10.3389/fimmu.2020.01708 (PMC7365923; doi:10.3389/fimmu.2020.01708)
Supplement: Supplementary Table 1 — Clinical trial registration for inflammatory disorder with COVID-19. [file Table_1.DOCX]

| **No.** | **Registered title** | **Registration ID** | **Intervention (ignore placebo and standard therapy)** | **Durg target (targeted medicine only)** | **Inclusion criteria (inflammatory disorder in COVID-19 only)** | **Participants (total)** |
| --- | --- | --- | --- | --- | --- | --- |
| 1 | **Early Identification and Treatment of Cytokine Storm Syndrome in Covid-19** | NCT04362111 | Anakinra | IL-1 receptor type 1 | 1.Hyperferritinemia (>700 ng/ml). Fever >38 degrees. 2.Any two of the following: a. Elevated d-dimer (>500 ng/ml) or thrombocytopenia (<130,000/mm3) b. elevated AST or ALT (> 2X ULN) c. elevated LDH (> 2X ULN) | 20 |
| 2 | **Defibrotide as Prevention and Treatment of Respiratory Distress and Cytokine Release Syndrome of Covid 19. (DEFACOVID)** | NCT04348383 | Defibrotide | NA | COVID-19 positive patients WHO grades 4, 5 or 6. (1) Grade 4: hospitalized requiring oxygen therapy. (2) Grade 5: hospitalized requiring high-flow oxygen therapy, noninvasive mechanical ventilation, or both. (3) Grade 6: hospitalized requiring ECMO, mechanical ventilation or both. | 120 |
| 3 | **Study of Efficacy and Safety of Canakinumab Treatment for CRS in Participants With COVID-19-induced Pneumonia (CAN-COVID)** | NCT04362813 | Canakinumab | IL-1 beta | 1.SpO_2_ <= 93% on room air or PaO_2_/ FiO_2_ < 300mmHg 2.CRP >=20 mg/L or ferritin level >=600 µg/L | 450 |
| 4 | **Etoposide in Patients With COVID-19 Infection** | NCT04356690 | Etoposide | NA | Evidence of cytokine storm defined as: Peak ferritin > 10,000 ng/mL OR Peak ferritin > 500 ng/mL and one or more of the following at any time during hospital admission: LDH > 500 U/L, d-dimer >1000 ng/mL, CRP > 100 mg/L, or white blood count> 15 k/microlitre Cohort 2: Intubated status as a result of COVID-19 infection-associated respiratory illness. Cohort 1 (if activated): Evidence of progressive respiratory failure (requiring >4 L/min of supplemental oxygen) without intubation; | 134 |
| 5 | **Use of the Interleukin-6 Inhibitor Clazakizumab in Patients With Life-threatening COVID-19 Infection** | NCT04363502 | Clazakizumab | Monoclonal antibody against IL-6 | Respiratory failure manifesting as: ARDS (defined by a P/F ratio of <200), OR SpO_2_ < 90% on 4L (actual or expected given higher O_2_ requirement) OR increasing O_2_ requirements over 24 hours, PLUS 2 or more of the following predictors for severe disease: CRP > 35 mg/L; Ferritin > 500 ng/mL; D-dimer > 1 mcg/L; Neutrophil-Lymphocyte Ratio > 4; LDH > 200 U/L; Increase in troponin in patient w/out known cardiac disease. | 30 |
| 6 | **A Randomized Placebo-controlled Safety and Dose-finding Study for the Use of the IL-6 Inhibitor Clazakizumab in Patients With Life-threatening COVID-19 Infection** | NCT04343989 | Clazakizumab | Monoclonal antibody against IL-6 | Respiratory failure manifesting as: ARDS (defined by a P/F ratio of <200), OR SpO_2_ < 90% on 4L (actual or expected given higher O2 requirement) OR increasing O_2_ requirements over 24 hours, PLUS 2 or more of the following predictors for severe disease: CRP > 35 mg/L Ferritin > 500 ng/mL D-dimer > 1 mcg/L Neutrophil-Lymphocyte Ratio > 4 LDH > 200 U/L Increase in troponin in patient w/out known cardiac disease. | 90 |
| 7 | **The Efficacy and Safety of Thalidomide Combined With Low-dose Hormones in the Treatment of Severe COVID-19** | NCT04273581 | Thalidomide | NA | 1. The laboratory (RT-PCR) confirmed the diagnosis of severe patients infected with CoVID-19 (refer to the fifth edition of the Chinese diagnosis and treatment guideline for trial); the diagnosis of new coronavirus pneumonia was confirmed, and any of the following: 1) Respiratory distress, breathing >=30 beats / min; 2) In the resting state, the SaO_2_ is <=93%; 3) Arterial blood oxygen partial pressure / oxygen concentration <=300mmHg | 40 |
| 8 | **Study of TJ003234 (Anti-GM-CSF Monoclonal Antibody) in Subjects With Severe Coronavirus Disease 2019 (COVID-19)** | NCT04341116 | TJ003234 | Anti-GM-CSF Monoclonal Antibody | Bilateral lung infection confirmed by imaging. Hospitalized patients >=60 years old with underlying medical comorbidities OR Severe disease that meets one of the following conditions: (i) At rest, finger blood SaO_2_ <= 93% or PaO_2_/FiO_2_ <= 300 mmHg; OR (ii) Requiring non-invasive or invasive mechanical ventilation. | 144 |
| 9 | **Phase 3 Study to Evaluate Efficacy and Safety of Lenzilumab in Hospitalized Patients With COVID-19 Pneumonia** | NCT04351152 | Lenzilumab | human colony stimulating factor 2, Anti-GM-CSF Monoclonal Antibody | Pneumonia diagnosed by Chest Xray, Computed Tomography or Magnetic Resonance imaging revealing infiltrates consistent with pneumonia and not yet developed ARDS | 238 |
| 10 | **Randomized Open-blind Controlled Trial to Study the Benefit of Colchicine in Patients With COVID-19 (COL-COVID)** | NCT04350320 | Colchicine | NA | Admitted in the hospital in the previous 48 hours, with clinical status 3, 4 or 5 of WHO classification. | 102 |
| 11 | **Colchicine Counteracting Inflammation in COVID-19 Pneumonia (ColCOVID-19)** | NCT04322565 | Colchicine | NA | Positive nasopharyngeal swab for COVID-19, asymptomatic or paucisymptomatic, aged >=70 years and/or with clinical risk factors for poor outcome (clinically relevant chronic lung disease, diabetes and/or heart disease) or symptomatic with respiratory or systemic symptoms, however clinically stable (MEWS<3) with CT imaging showing viral pneumonia and positive or pending pharyngo-nasal swab for COVID-19: Temperature 38°C and/or intensive cough, RR < 25 /min, SaO_2_ (pulse oximetry) >95% with respiratory and/or systemic symptoms and initial mild respiratory failure e with objective signs of lung involvement; the patient is in stable conditions (MEWS < 3) Temperature>38°C and or intensive cough, RR >=25 /min, or SaO_2_ 94- 95% in room air | 310 |
| 12 | **Colchicine Twice Daily During 10 Days as an Option for the Treatment of Symptoms Induced by Inflammation in Patients With Mild and Severe Coronavirus Disease (ColchiVID)** | NCT04367168 | Colchicine | NA | Diagnosed with COVID-19 with mild or severe disease | 174 |
| 13 | **Assessment the Activity Value of 13- Cis-Retinoic Acid(Isotretinoin) in the Treatment of COVID-19 (Isotretinoin)** | NCT04353180 | Isotretinoin (13 cis retinoic acid ) | NA | Adult SARI patients with 2019-ncov infection confirmed by PCR; Absolute value of lymphocytes < 0. 6x 10^9^/L; Severe respiratory failure within 48 hours and requires admission to ICU. (severe respiratory failure was defined as PaO_2_/FiO_2_ < 200 mmHg and was supported by positive pressure mechanical ventilation (including non-invasive and invasive mechanical ventilation, PEEP>=5cmH_2_O)) | 45 |
| 14 | **Chloroquine Outpatient Treatment Evaluation for HIV-Covid-19 (CQOTE)** | NCT04360759 | Chloroquine or hydroxychloroquine | NA | Not requiring immediate hospitalisation; Mild disease, defined as RR <25/min, pulse rate <120/min, SpO_2_ >94%; HIV-positive by rapid test or documented history; Suspected or confirmed Covid-19. | 560 |
| 15 | **Hydroxychloroquine Versus Placebo in COVID-19 Patients at Risk for Severe Disease (HYCOVID)** | NCT04325893 | Hydroxychloroquine | NA | Having at least one of the following two risk factors for complications: age >=75 years old SpO_2_ <= 94% while breathing ambient air, or a PaO_2_ to FiO_2_ ratio <= 300 mmHg. Patients affiliated with or benefitting from a social security scheme Written and signed consent of the patient or a relative or, if not possible, emergency inclusion procedure Electrocardiogram showing absence of QT prolongation greater than 440 ms in men and 460 ms in women. | 1300 |
| 16 | **A Pilot Study to Assess Hydroxychloroquine in Patients With SARS-CoV-2 (COVID-19)** | NCT04363866 | Hydroxychloroquine | NA | Must meet at least one of the following clinical stratifications: a. Have at least 1 minor criterion per ATS criteria (refer to Appendix A), or b. Have fever, respiratory symptoms, with pneumonia visible on chest imaging (e.g., X-ray or computed tomography [CT]), or c. High risk for poor outcome, as defined by any one of the following: i. Age >=60 years old. Cardiovascular disease. Diabetes. Chronic respiratory disease (e.g., COPD). Hypertension, defined as blood pressure >=140 / 90 mmHg. iii. Solid organ or stem cell transplant recipient. iv. Diagnosis of solid or hematologic malignancy being treated with systemic chemotherapy. v. Receipt of biologic agent or prednisone > 0.5 mg/kg/day (or equivalent). | 40 |
| 17 | **Hydroxychloroquine as Chemoprevention for COVID-19 for High Risk Healthcare Workers** | NCT04345653 | Hydroxychloroquine Sulfate | NA | Considered high-risk healthcare care providers in a hospital setting with active exposure to COVID-19 infection. High-risk HCP's are defined as those actively working during the study duration in the Emergency Department and in the Intensive Care Setting, for the purpose of this study. | 45 |
| 18 | **Immune Monitoring of Prophylactic Effect of Hydroxychloroquine in Healthcare Providers Highly Exposed to COVID-19 (Chloroquine UN)** | NCT04346329 | Hydroxychloroquine | NA | Be part of the health personnel (intensivist doctor, hospital doctors, nurses, physical therapists and support staff in the care unit) who will work at the HUN in the period of May 1st, 2020 to August 1, 2020. Be over 18 years old, exposed to patients with COVID-19. Not having symptoms compatible with an acute respiratory infection for the last 21 days. | 86 |
| 19 | **Double Therapy With IFN-beta 1b and Hydroxychloroquine** | NCT04350281 | Group 1: Interferon Beta-1B; Group 2: Hydroxychloroquine | NA | COVID-19 | 80 |
| 20 | **Evaluation of Efficacy of Levamisole and Formoterol+Budesonide in Treatment of COVID-19** | NCT04331470 | Group 1: Levamisole Pill + Budesonide+Formoterol, inhaler;  Group 2: Lopinavir/Ritonavir + hydroxychloroquine | NA | Definitely positive COVID-19 patients | 30 |
| 21 | **Randomized Comparison of Combination Azithromycin and Hydroxychloroquine vs. Hydroxychloroquine Alone for the Treatment of Confirmed COVID-19** | NCT04336332 | Group 1: Hydroxychloroquine Sulfate + Azithromycin;  Group 2: Hydroxychloroquine Sulfate | NA | COVID-19 | 160 |
| 22 | **A Randomized, Open-label, Parallel, Controlled Trial for Evaluation of the Efficacy and Safety of Chloroquine Phosphate in the treatment of Severe Patients with Novel Coronavirus Pneumonia (COVID-19)** | ChiCTR2000029898 | Group 1: Hydroxychloroquine;  Group 2: Phosphate chloroquine | NA | Severe COVID-19 | 100 |
| 23 | **Clinical Study of Chloroquine Phosphate in the Treatment of Severe Novel Coronavirus Pneumonia (COVID-19)** | ChiCTR2000029988 | Chloroquine Phosphate | NA | Severe patients (in any case: respiratory distress, RR >= 30 times / min; in resting state, SaO_2_ <= 93%; PaO_2_ / FiO_2_ <= 300MMHG); Within 12 days after illness onset. | 80 |
| 24 | **A prospective, randomized, open label, controlled trial for chloroquine and hydroxychloroquine in patients with severe novel coronavirus pneumonia (COVID-19)** | ChiCTR2000029992 | Group 1: Chloroquine;  Group 2: Hydroxychloroquine | NA | SaO_2_ / SpO_2_ <= 94% or PaO_2_ / FiO_2_ ratio < 300mghg at room temperature at admission; Onset <=12 days. | 100 |
| 25 | **Study of Immune Modulatory Groups and Other Treatments in COVID-19 Patients: Sarilumab, Azithromycin, Hydroxychloroquine Trial - CORIMUNO-19 - VIRO (CORIMUNO-VIRO)** | NCT04341870 | Group 1: Sarilumab;  Group 2: Azithromycin;  Group 3: Hydroxychloroquine | Sarilumab: IL-6 receptor subunit alpha | COVID-19 cases not requiring ICU at admission with moderate or severe pneumopathy according to the WHO Criteria of severity of COVID pneumopathy: Moderate cases: Cases meeting all of the following criteria: [Showing fever and respiratory symptoms with radiological findings of pneumonia] AND [Requiring between 3L/min and 5L/min of oxygen to maintain SpO_2_>97%] OR Severe cases: Cases meeting any of the following criteria: [Respiratory distress ( >=30 breaths/ min)] OR [SaO_2_<=93% at rest in ambient air; or SaO_2_ <=97 % with O2 > 5L/min] OR [PaO_2_/FiO_2_ <= 300mmHg] | 60 |
| 26 | **Efficacy and Safety of Emapalumab and Anakinra in Reducing Hyperinflammation and Respiratory Distress in Patients With COVID-19 Infection.** | NCT04324021 | Group 1:Emapalumab;  Group 2: Anakinra | Emapalumab: Interferon gamma;  Anakinra: IL-1 receptor type 1 | 1. Presence of respiratory distress, defined as: a. PaO_2_/FiO_2_ < 300 mm Hg and >200 mm Hg or b. RR >=30 breaths/min or c. SpO_2_ < 93 percent in air at rest. Note: Patients given continous positive airway pressure (CPAP) ventilator support are eligible for inclusion.  2. Presence of hyperinflammation defined as: a. Lymphocyte counts < 1000 cells/µL, and b. Two of the following three criteria: i. Ferritin > 500ng/mL ii. LDH > 300 U/L iii. D-Dimers > 1000 ng/mL | 54 |
| 27 | **Dexmedetomidine to Improve Outcomes of ARDS in Critical Care COVID-19 Patients (COVID-DEX)** | NCT04358627 | Dexmedetomidine | NA | Patients with respiratory insufficiency criteria candidates to non-invasive ventilation techniques (high flow oxygen masks, non-invasive mechanical ventilation); SpO_2_ (at FiO_2_: 0.21) <= 93% equivalent to SaO_2_/FiO_2_<=442; PaO_2_/ FiO_2_ < 300; Bilateral opacities consistent with pulmonary edema must be present and may be detected on CT or chest radiograph that must not be fully explained by cardiac failure or fluid overload, in the physician’s best estimation using available information | 80 |
| 28 | **suPAR-guided Anakinra Treatment for Validation of the Risk and Management of Respiratory Failure by COVID-19 (SAVE) (SAVE)** | NCT04357366 | Group 1:Anakinra,  Group 2: trimethoprim/sulfamethoxazole | Anakinra: IL-1 receptor type 1 | Plasma suPAR >=6ng/ml | 100 |
| 29 | **Evaluation of Activity and Safety of Oral Selinexor in Participants With Severe COVID-19 Infection (Coronavirus)** | NCT04349098 | Selinexor | NA | Has symptoms of severe COVID-19 as demonstrated by: At least one of the following: fever, cough, sore throat, malaise, headache, muscle pain, shortness of breath at rest or with exertion, confusion, or symptoms of severe lower respiratory symptoms including dyspnea at rest or respiratory distress AND Clinical signs indicative of lower respiratory infection with COVID-19, with at least one of the following: RR >=30 breaths/minute (min), heart rate >=125 /min, SaO2 < 93% on room air or requires > 2 Liter (L)/minute oxygen by NC in order maintain SaO2 >=93%, PaO_2_/FiO_2_ < 300 mm/hg. Concurrent anti-viral and/or anti-inflammatory agents (e.g., biologics, hydroxychloroquine) are permitted. | 230 |
| 30 | **TOFAcitinib in SARS-CoV2 Pneumonia** | NCT04332042 | Tofacitinib | JAK1 and JAK3 | Rx or CT-scan confirmed interstitial pneumonia. Hospital admission from less than 24h. | 50 |
| 31 | **Ruxolitinib for the Treatment of Acute Respiratory Distress Syndrome in Patients With COVID-19 Infection (RESPIRE)** | NCT04361903 | Ruxolitinib | JAK1 and JAK2 | Maging (CT / ECO / RX) positive for pneumonia; SaO_2_ of 93% or less in the environment; PaO_2_ on FiO_2_ (PaO_2_ / FiO_2_) lower than 250 mg / Hg, but not lower than 100 mg / Hg; Rapid clinical evolution with worsening of respiratory parameters in the last 12 hours. | 13 |
| 32 | **Ruxolitinib for Treatment of Covid-19 Induced Lung Injury ARDS (RuXoCoil)** | NCT04359290 | Ruxolitinib | JAK1 and JAK2 | Severe lung disease as defined by following: a. Recent intubation; b. Requirement of invasive ventilation moderate to severe pulmonary oxygen exchange disturbance as defined by (PaO_2_/FiO_2_) <= 200 mmHg at a PEEP >=5mm H_2_O; c. Serum LDH > 283 U/l; d. Ferritin above normal value;e. CT-scan: pulmonary infiltration compatible with Covid-19 disease. | 15 |
| 33 | **Safety and Efficacy of Ruxolitinib for COVID-19** | NCT04348071 | Ruxolitinib | JAK1 and JAK2 | Illness of any duration that meets each of the following: | 80 |
| 34 | **Expanded Access Program of Ruxolitinib for the Emergency Treatment of Cytokine Storm From COVID-19 Infection** | NCT04355793 | Ruxolitinib | JAK1 and JAK2 | Disease severity making the patient eligible for hospitalization (whether or not hospitalization is available), with evidence of cytokine storm as determined by the treating physician. Manifestations of cytokine storm can include the following: Severe shortness of breath (RR > 24 breaths/minute). SpO_2_ of < 90% on ambient air. Need for invasive or noninvasive mechanical ventilation. ARDS. Multiple organ failure. |  |
| 35 | **Ruxolitinib to Combat COVID-19** | NCT04354714 | Ruxolitinib | JAK1 and JAK2 | A diagnosis of advanced COVID-19 as defined by both of the following: A positive test for SARS-CoV-2 RNA detected by RT-PCR collected from the upper respiratory tract (nasopharyngeal and oropharyngeal swab) and, if possible, the lower respiratory tract (sputum, tracheal aspirate, or bronchoalveolar lavage), analyzed by a CLIA certified lab. Critical disease manifested by any of the following: Chest imaging (CT or chest X-ray permitted) with >=50% lung involvement. Respiratory failure requiring invasive mechanical ventilation or supplementary oxygen with FiO2 >=50%. Shock (defined as mean arterial pressure <= 65 mmHg unresponsive to 25ml/kg isotonic intravenous fluid resuscitation and/or requiring vasopressor support. Cardiac dysfunction defined by: New global systolic dysfunction with ejection fraction <= 40%. Takotsubo cardiomyopathy. New onset supraventricular or ventricular arrhythmias. Plasma troponin I >=0.10 ng/mL in someone without previously documented troponin elevation beyond that level. Elevated plasma NT-proBNP in someone without documented prior elevation. If Age < 50, NT-proBNP > 450 pg/ml. If Age 50-74, NT-proBNP > 900 pg/ml. If Age >=74, NT-proBNP > 1800 pg/ml. If Age >=74, NT-proBNP > 1800 pg/ml. Adequate hematologic function defined as: absolute neutrophil count >=1000/mm3. Platelet count >=50,000/mm3 without growth factor or transfusion support for 7 days prior to screening. Creatinine clearance >=15 mL/minute or receiving renal replacement therapy. | 25 |
| 36 | **Phase 3 Randomized, Double-blind, Placebo-controlled Multi-center Study to Assess the Efficacy and Safety of Ruxolitinib in Patients With COVID-19 Associated Cytokine Storm (RUXCOVID) (RUXCOVID)** | NCT04362137 | Ruxolitinib | JAK1 and JAK2 | Patients with coronavirus (SARS-CoV-2) infection confirmed by polymerase chain reaction (PCR) test or another rapid test from the respiratory tract prior to randomization. Patients currently hospitalized or will be hospitalized prior to randomization. Patients with lung imaging showing pulmonary infiltrates (chest X-ray or CT scan) prior to randomization. Patients, who meet at least one of the below criteria: Respiratory frequency >=30/min; SaO_2_ <= 93% on room air; PaO_2_/ FiO_2_ < 300mmHg (1mmHg=0.133kPa) (corrective formulation should be used for higher altitude regions (over 1000m). | 402 |
| 37 | **Treatment of SARS Caused by COVID-19 With Ruxolitinib** | NCT04334044 | Ruxolitinib | JAK1 and JAK2 | Increase in work of breathing or presence of dyspnea | 20 |
| 38 | **Ruxolitinib in Covid-19 Patients With Defined Hyperinflammation (RuxCoFlam)** | NCT04338958 | Ruxolitinib | JAK1 and JAK2 | Patients with temperature > 37.3°C. Patients with respiratory symptoms and/or hypoxia SpO2 < 93%. Patients with Covid-19 stage II and stage III. Patients, with a Covid Inflammation Score >=10. | 200 |
| 39 | **Study of the Efficacy and Safety of Ruxolitinib to Treat COVID-19 Pneumonia** | NCT04331665 | Ruxolitinib | JAK1 and JAK2 | Need for supplemental oxygen to maintain SaO_2_ > 93% | 64 |
| 40 | **Ruxolitinib Managed Access Program (MAP) for Patients Diagnosed With Severe/Very Severe COVID-19 Illness** | NCT04337359 | Ruxolitinib | JAK1 and JAK2 | Adult and adolescent patients (>=12years), who meet one of the below criteria. Respiratory frequency >=30/min. SaO_2_ <= 93% on room air (FiO_2_=0.21). PaO_2_/FiO_2_ <300mmHg(1mmHg=0.133kPa) (corrective formulation should be used for higher altitude regions (over 1000m).Pediatric patients (>=6-<12 years) who meet one of the below criteria (where appropriate): Shortness of breath. SaO_2_ <92% on room air (Fi)2=0.21). Labored breathing (e.g. wheezing, flaring of nostrils, three concave sign), cyanosis, intermittent apnea. Lethargy or convulsions. Refusal to eat or difficulty with feeding; signs of dehydration. |  |
| 41 | **Ruxolitinib for the Treatment of Acute Respiratory Distress Syndrome in Patients With COVID-19 Infection (RESPIRE)** | NCT04361903 | Ruxolitinib | JAK1 and JAK2 | Inclusion Criteria: 1. Imaging (CT / ECO / RX) positive for pneumonia; 2. SaO_2_ of 93% or less in the environment; 3. PaO_2_ on FiO_2_ (PaO_2_ / FiO_2_) lower than 250 mg / Hg, but not lower than 100 mg / Hg; 4. Rapid clinical evolution with worsening of respiratory parameters in the last 12 hours. | 13 |
| 42 | **Ruxolitinib for Treatment of Covid-19 Induced Lung Injury ARDS (RuXoCoil)** | NCT04359290 | Ruxolitinib | JAK1 and JAK2 | Severe lung disease as defined by following: 1. Recent intubation; 2. Requirement of invasive ventilation moderate to severe pulmonary oxygen exchange disturbance as defined by (PaO_2_/FiO_2_) ≤ 200 mmHg at a PEEP ≥ 5mm H2O; 3. Serum LDH > 283 U/l; 4. Ferritin above normal value; 5. CT-scan: pulmonary infiltration compatible with Covid-19 disease | 15 |
| 43 | **Efficacy of Intravenous Anakinra and Ruxolitinib During COVID-19 Inflammation (JAKINCOV) (JAKINKOV)** | NCT04366232 | Group 1: Anakinra alone;  Group 2: Anakinra and Ruxolitinib | Anakinra: IL-1 receptor type 1. Ruxolitinib: JAK1 and JAK2. | Patient hospitalized with clinical, biological and radiological features corresponding to the following stages : Stage 2b: hypoxic pneumonia (respiratory frequency > 30/mn, SaO_2_ < 90 mmHg on room air) associated with a clear biological inflammatory syndrome (CRP > 150 mg/l). Stage 3: ARDS defined by a patient under mechanical ventilation with a ratio PaO_2_/FiO_2_ < 300 for more than 24h. Evolved stage 3: ARDS according to previous definition associated with another organ failure or syndrome among: A state of shock with noradrenaline dosing > 3mg/h. Acute kidney failure oligo-anuric or justifying extra-renal purification. Hepatocellular insufficiency or coagulopathy with a V factor < 50%. Myocarditis responsible for acute heart failure and or cardiogenic shock. Hemophagocytic syndrome. Hyperferritinemia > 5000 ng/mL. | 50 |
| 44 | **Study of Ruxolitinib Plus Simvastatin in the Prevention and Treatment of Respiratory Failure of COVID-19. (Ruxo-Sim-20)** | NCT04348695 | Ruxolitinib plus simvastatin | Ruxolitinib: JAK1 and JAK2 | Clinical diagnosis or confirmed by analytical tests (PCR of viral RNA or detection of antiSARS-Cov-2 antibodies) that requires care in hospital and that are grade 3 or 4 of the WHO 7-point ordinal scale of severity categorization for COVID. Platelets> 50,000 / uL, neutrophils> 500 / ul Kidney or liver failure is not a contraindication, dose adjustment will be made according to the SmPC | 94 |
| 45 | **COVID-19: A Pilot Study of Adaptive Immunity and Anti-PD1** | NCT04356508 | Nivolumab | Programmed cell death protein 1 | Clinically stable with disease severity defined as mild or moderate (mild disease is defined as symptoms with or without lung infiltrates on chest X-Ray or CT imaging; moderate disease is defined as lung infiltrates with evidence of type 1 respiratory failure). Asymptomatic patients may be enrolled if patients have obvious radiographic changes on chest or CT radiography deemed to be related to COVID-19. | 15 |
| 46 | **Efficacy and Safety of Tocilizumab in the Treatment of SARS-Cov-2 Related Pneumonia (TOSCA)** | NCT04332913 | Tocilizumab | IL-6 receptor subunit alpha | Patients with SARS-CoV-2 infection confirmed by tests (RT-PCR) and pulmonary involvement, hospitalized, at the end of the initial phase of high viral load of COVID-19 (apyretic> 72 hours and / or at least 7 days after onset of symptoms); Worsening of respiratory exchanges such as to require non-invasive or invasive ventilation support (BCRSS score >=3). High levels of IL-6 (> 40 pg/mL) or alternatively CRP and/or ferritin and/or D-dimer and/or fibrinogen values higher than the reference values or rapidly increasing; | 30 |
| 47 | **Tocilizumab in the Treatment of Coronavirus Induced Disease (COVID-19) (CORON-ACT)** | NCT04335071 | Tocilizumab | IL-6 receptor subunit alpha | CRP >=50mg/L plus 3 out of the following 5 criteria need to be fulfilled: RR >=25, SpO_2_ <93% (on ambient air), PaO_2_ <65 mmHg, Persistent or increasing dyspnoea as defined by a one point increase on the mMRC dyspnoea scale (over 1 hour), Persistent or increasing oxygen demand (over 1 hour). | 100 |
| 48 | **Tocilizumab for SARS-CoV2 (COVID-19) Severe Pneumonitis** | NCT04315480 | Tocilizumab | IL-6 receptor subunit alpha | CT-scan confirmed multifocal interstitial pneumonia; Need of oxygen therapy to maintain SO2>93%; Worsening of lung involvement, defined as (one of the following criteria): Worsening of SaO_2_ >3 percentage points or decrease in PaO_2_ >10%, with stable FiO_2_ in the last 24h; Need of increase FiO_2_ in order to maintain a stable SO_2_ or new onset need of mechanical ventilation in the last 24h; Increase in number and/or extension of pulmonary areas of consolidation. | 38 |
| 49 | **Tocilizumab vs CRRT in Management of Cytokine Release Syndrome (CRS) in COVID-19 (TACOS)** | NCT04306705 | Tocilizumab | IL-6 receptor subunit alpha | 1. Illness of any duration, and at least one of the following:a. Radiographic infiltrates by imaging (chest x-ray, CT scan, etc.), OR b. Clinical assessment (evidence of rales/crackles on physical examination) AND SpO_2_ <=93% on room air, OR c. Requiring mechanical ventilation and/or supplemental oxygen, OR d. Sustained fever in the past 24 hours and unresponsive to NSAID or steroid 2. Serum IL-6 >=3 times the upper limit of normal | 120 |
| 50 | **Tocilizumab for the Treatment of Cytokine Release Syndrome in Patients With COVID-19 (SARS-CoV-2 Infection)** | NCT04361552 | Tocilizumab | IL-6 receptor subunit alpha | Should be hospitalized and exhibit at least one of the following predictors of mortality: Age >= 65 years; Current smoker (smoked >= 100 cigarettes in life and actively smoking); Chronic obstructive pulmonary; disease; Diabetes; Hypertension; Coronary artery disease; Cerebrovascular accident (CVA); Chronic renal disease (creatinine of >= 2 mg/dl); Cancer; Patients that have CRP >= 10 mg/L; D-dimer >= 0.5 mg/L; Procalcitonin >= 0.5 mg/L; Lactate dehydrogenase >= ULN | 180 |
| 51 | **Treatment of COVID-19 Patients With Anti-interleukin Groups (COV-AID)** | NCT04330638 | Group 1: Anakinra;  Group 2: Siltuximab;  Group 3: Tocilizumab | Anakinra:IL-1 receptor type 1, Siltuximab: IL-6, Tocilizumab: IL-6 receptor subunit alpha | 1. Presence of hypoxia defined as PaO_2_/FiO_2_ below 350 while breathing room air in upright position or PaO_2_/FiO_2_ below 280 on supplemental oxygen and immediately requiring high flow oxygen device or mechanical ventilation. 2. signs of cytokine release syndrome defined as ANY of the following: 1) serum ferritin concentration >1000 mcg/L and rising since last 24h; 2) single ferritin above 2000 mcg/L in patients requiring immediate high flow oxygen device or mechanical ventilation; 3) lymphopenia defined as <800 lymphocytes/microliter) and two of the following extra criteria: a. Ferritin > 700 mcg/L and rising since last 24h; b. increased LDH (above 300 IU/L) and rising last 24h; c. D-Dimers > 1000 ng/mL and rising since last 24h; d. CRP above 70mg/L and rising since last 24h and absence of bacterial infection; e. if three of the above are present at admission, no need to document 24h rise. | 342 |
| 52 | **Personalised Immunotherapy for SARS-CoV-2 (COVID-19) Associated With Organ Dysfunction (ESCAPE)** | NCT04339712 | Group 1: Anakinra,  Group 2: Tocilizumab | Anakinra: IL-1 receptor type 1.  Tocilizumab: IL-6 receptor subunit alpha. | Organ dysfunction defined as the presence of at least one of the following conditions: Total SOFA score greater than or equal to 2; Involvement of the lower respiratory tract; Laboratory documentation of MAS or immune dysregulation. MAS is documented by the findings of any serum ferritin greater than 4,420ng/ml. immune dysregulation is documented by the combination of two findings: a) serum ferritin equal to or lower than 4,420ng/ml; and b) less than 5,000 receptors of the membrane molecule of HLA-DR on the cell membrane of blood CD14-monocytes or less than 30 MFI of HLA-DR on the cell membrane of blood CD14-monocytes as counted by flow cytometry. | 40 |
| 53 | **Clinical Trial of Sarilumab in Adults With COVID-19 (SARICOR)** | NCT04357860 | Sarilumab (Kevzara) | IL-6 receptor subunit alpha | Admission for confirmed respiratory symptoms to COVID-19 based on a positive PCR in a sample of the respiratory tract in the local laboratory in the absence of respiratory distress syndrome requiring ONAF or mechanical ventilation; IL-6 levels> 40 pg/ml. In its absence, D-Dimer > 1500 or> 1000 may be included if progressive increases are documented. | 120 |
| 54 | **Low Dose of IL-2 In Acute Respiratory DistrEss Syndrome Related to COVID-19 (LILIADE-COVID)** | NCT04357444 | ILT101 | IL-2 | Patient is intubated and mechanically ventilated; Diagnosis of ARDS according to the Berlin definition of ARDS; Onset of ARDS <96 hours. | 30 |
| 55 | **Study to Evaluate the Efficacy and Safety of Tocilizumab Versus Corticosteroids in Hospitalised COVID-19 Patients With High Risk of Progression** | NCT04345445 | Group 1:Tocilizumab;  Group 2:Methylprednisolone | Tocilizumab: IL-6 receptor subunit alpha | Presence of clinical and radiological signs of progressive disease, AND laboratory evidence indicative of risk for cytokine storm complications:Clinical: Dyspnoea OR RR>20 breaths/min AND O2 sat <93% on RA OR increasing need for O2 supplementation to maintain O2 sat >95% on RA WITH Radiological: CXR or CT indicative of pneumonia OR worsening findings over time AND Laboratory: CRP levels >60 OR an increase of CRP >20 over 12 hours WITH an increasing ferritin level OR declining lymphocyte counts | 310 |
| 56 | **Anti-il6 Treatment of Serious COVID-19 Disease With Threatening Respiratory Failure (TOCIVID)** | NCT04322773 | Group 1: RoActemra;  Group 2: Kevzara | RoActemra: IL-6 receptor subunit alpha.  Kevzara: IL-6 receptor subunit alpha | Need of oxygen therapy to maintain SO2>94% OR FiO2/PaO2 > 20 and at least two of the following laboratory measures: CRP level >70 mg/L, CRP level >= 40 mg/L and doubled within 48 hours (without other confirmed infectious or non-infectious course), Lactatdehydrogenase > 250 U/L, thrombocytopenia < 120.000 x 10E9/L, lymphocyte count < 0.6 x 10E9/L, D-dimer > 1 ug/mL, serum ferritin > 300 ug/mL | 200 |
| 57 | **A Study of CM4620-Injectable Emulsion (IE) in Patients With Severe COVID-19 Pneumonia** | NCT04345614 | CM4620-Injectable Emulsion | calcium-release activated calcium-channel (CRAC channel) inhibitor | 1. At least 1 of the following symptoms: Fever, cough, sore throat, malaise, headache, muscle pain, dyspnea at rest or with exertion, confusion, or respiratory distress; 2. At least 1 of the following clinical signs; RR >=30, heart rate >=125, arterial SaO_2_ <93% on room air or requires >2 liters oxygen by nasal cannula to maintain SaO_2_ >=93%, or PaO_2_/FiO_2_ <300, estimated from pulse oximetry or determined by arterial blood gas; 3. The presence of a respiratory infiltrate or abnormality consistent with pneumonia that is documented by either a chest X-ray or computerized tomography scan of the lungs. | 120 |
| 58 | **The Fleming [FMTVDM] Directed CoVid-19 Treatment Protocol (FMTVDM)** | NCT04349410 | Group 1: Hydroxychloroquine, Azithromycin  Group 2: Hydroxychloroquine, Doxycycline  Group 3: Hydroxychloroquine, Clindamycin  Group 4: Hydroxychloroquine, Clindamycin, Primaquine - low dose.  Group 5: Hydroxychloroquine, Clindamycin, Primaquine - high dose.  Group 6: Remdesivir  Group 7: Tocilizumab  Group 8: Methylprednisolone  Group 9: Interferon-Alpha2B  Group 10: Losartan  Group 11: Convalescent Serum | Tocilizumab: IL-6 receptor subunit alpha. | COVID-19 | 500 |
| 59 | **A clinical study for the efficacy and safety of Adalimumab Injection in the treatment of patients with severe novel coronavirus pneumonia (COVID-19)** | ChiCTR2000030089 | Adalimumab | TNF | In accordance with NCP criteria for severe and critical illness, namely "Pneumonitis Diagnosis and Treatment Scheme for Novel Coronavirus Infection (Trial Version 6)". | 60 |
| 60 | **Efficacy and safety of adamumab combined with tozumab in severe and critical patients with novel coronavirus pneumonia (COVID-19)** | ChiCTR2000030580 | Tocilizumab combined with adalimumab (Qletli) | Tocilizumab: IL-6 receptor subunit alpha.  Adalimumab: TNF | Severe (including critical risk factors) and critical patients with confirmed novel coronavirus pneumonia; Blood CRP and ESR were more than twice higher than normal or TNF-α and IL-6 were higher than normal. | 60 |
| 61 | **Study for safety and efficacy of Jakotinib hydrochloride tablets in the treatment severe and acute exacerbation patients of novel coronavirus pneumonia (COVID-19)** | ChiCTR2000030170 | Jakotinib hydrochloride tablets | JAK1 | Remarks: “Judgment Criteria for NCP Patients with Acute Exacerbation in General to Severe and Critical Severity” 1) Aged 50 years or older; 2) Dyspnea (those who inhale oxygen in a mask); 3) Combined with basic diseases, such as COPD, diabetes, hypertension, coronary heart disease; 4) White blood cell count is less than 4 × 109 / L or has a tendency to decrease continuously; 5) Lymphocyte count <1.5x10^9 / L or a trend of continuous decrease; 6) Platelet count is less than 150x10^9/L or has a tendency to decrease continuously; 7) Hemoglobin has continued to decrease; 8) CRP >=10 mg / L or a trend of continuous increase; 9) Procalcitonin >=0.5 ng / mL or a tendency of continuous increase; 10) LDH >=250 U/L or a trend of continuous increase; 11) Aspartate aminotransferase and alanine aminotransferase have a tendency to increase continuously; 12) Creatinine >=133 umol/L or a tendency of continuous increase; 13) D-dimer >=0.5 mg/L or a trend of continuous increase; 14) There is a continuous decrease in serum potassium; 15) IL-6 has a tendency to increase continuously; 16) Any of IL-2, IL-4, IL-10, TNF, IFN-r, and IL-17A has a tendency to increase continuously; 17) Imaging findings consistent with NCP progression. Remarks: (1) If 9 or more of the above 17 items are met, they are judged to be “normal-to-severe, severely severe NCP patients with acute exacerbation period”; (2) continuous increase or decrease trend: at least 3 consecutive measurements, specific trends Determined by the research doctor; (3) In view of the special circumstances of the NCP, during the trial implementation, the selection criteria of the protocol can be appropriately updated and adjusted according to the clinical progress. | 16 |
| 62 | **Pyridostigmine in Severe SARS-CoV-2 Infection (PISCO)** | NCT04343963 | Pyridostigmine Bromide | NA | Need for hospitalization with increased mortality criteria according to published observations, including one or more of the following severity criteria according to the treating medical team: to. Dyspnoea b. Lung infiltrates> 50% of lung fields by CT c. A ratio of PaO_2_ to the FiO_2_ <300 mmHg d. Pulse oximetry <90% to ambient air, or a 3% drop in baseline oximetry, or need to increase supplemental oxygen due to chronic hypoxia, as well as the need for supplemental oxygen according to medical judgment and. Alteration of one or more of the following laboratory studies at the time of hospital admission: i. D-dimer >1 ug/mL ii. Ferritin level >300 ng/mL iii. CRP >3mg/L iv. LDH >245 U/L v. Lymphopenia <800 cells/uL vi. Creatine kinase (CK) level >800 IU/L | 436 |
| 63 | **Prophylactic Corticosteroid to Prevent COVID-19 Cytokine Storm** | NCT04355247 | Methylprednisolone | NA | Meet high risk criteria | 20 |
| 64 | **Corticosteroids During Covid-19 Viral Pneumonia Related to SARS-Cov-2 Infection (CORTI-Covid)** | NCT04344288 | Prednisone | NA | Peripheral saturation by pulse oximeter SpO2 <= 94% in ambient air measured twice at 5-15 min intervals, or PaO2 / FiO2 <300 mmHg, | 304 |
| 65 | **Clinical Trial to Evaluate Methylprednisolone Pulses and Tacrolimus in Patients With COVID-19 Lung Injury (TACROVID)** | NCT04341038 | Group 1: Tacrolimus, Group 2: Methylprednisolone | NA | Respiratory failure (PaO2 / FiO2 <300 or satO2 / FiO2 <220); PCR>100 mg/L and/or D-Dimer>1000 µg/L and/or Ferritin>1000 ug/L | 84 |
| 66 | **Adjunctive Corticosteroid Therapy for Patients with Severe Novel Coronavirus Pneumonia (COVID-19): a Randomized Controlled Trial** | ChiCTR2000029386 | Glucocorticoid | NA | The diagnosis of severe coronavirus pneumonia will have to meet at least one of the following criteria: (1) Respiratory distress, RR>30 times/minute (2) In the state of no oxygen at rest, the patient’s SPO2<=93% (3) Oxygenation Index (PaO2/FiO2)<=300 mmHg (1mmHg=0.133kPa); (4) Respiratory failure requiring mechanical ventilation; (5) Sepsis; (6) Other organ failure requiring ICU care. | 48 |
| 67 | **Exploratory study for Immunoglobulin From Cured COVID-19 Patients in the Treatment of Acute Severe novel coronavirus pneuvirus (COVID-19)** | ChiCTR2000030841 | Immunoglobulin From Cured COVID-19 Patients | NA | Patients diagnosed with acute severe 2019-nCoV pneumonia: (1) Laboratory (RT-PCR) confirmed infection with 2019-nCoV. (2) Lung involvement confirmed with pulmonary CT scan; (3) At least one of the following conditions should be met: respiratory distress, RR >=30 times/min; SaO_2_ <=93% in resting state; PaO2/FiO2 <=300mmHg; respiratory failure and mechanical ventilation are required; shock occurs; ICU monitoring and treatment is required in combination with other organ failure. | 10 |
| 68 | **Anti COVID-19 Convalescent Plasma Therapy** | NCT04345679 | anti-SARS-CoV-2 convalescent plasma | NA | Admitted to hospital due to SARS CoV-2 infection | 20 |
| 69 | **Bone Marrow-Derived Mesenchymal Stem Cell Treatment for Severe Patients With Coronavirus Disease 2019 (COVID-19)** | NCT04346368 | Bone Marrow-Derived MSCs (BM-MSCs) | NA | Clinical classification is severe case: Meet any of the following: 1) Increased RR (>=30 beats / min), difficulty breathing, cyanosis of the lips; 2) SpO_2_ <=93% at rest ; 3) PaO_2_ / FiO_2_ <=300 mmHg (1mmHg = 0.133kPa). | 20 |
| 70 | **Safety and Efficacy Study of Allogeneic Human Dental Pulp Mesenchymal Stem Cells to Treat Severe COVID-19 Patients** | NCT04336254 | Allogeneic human dental pulp stem cells (BSH BTC & Utooth BTC) | NA | Diagnosed with severe pneumonia of COVID: respiratory distress, RR >30 times / min; resting SaO_2_ of 93% or less; arterial partial pressure of oxygen / oxygen concentration 300mmHg; SARS-CoV-2 nucleic acid test was positive. | 20 |
| 71 | **ASC Therapy for Patients With Severe Respiratory COVID-19 (ASC COVID-19)** | NCT04341610 | Stem Cell Product | NA | Pulmonary symptoms and signs, at least one of the following before clinical decision for intubation and respirator treatment: 1. Respiratory distress, RR >=30/min; 2. SaO_2_ <= 93% at rest state; 3. PaO_2_ / FiO_2_ <= 300mmHg, 1mmHg=0.133kPa; In respirator and possible for treatment within the first 24 hours. | 40 |
| 72 | **Compassionate Use Open-Label Anti-CD14 Treatment in Patients With SARS-CoV-2 (COVID-19)** | NCT04346277 | Biological: IC14, a monoclonal antibody against CD14 | CD14 | Hypoxemia as defined by any of the following: a. SpO_2_ <=92% on room air; b. Requirement for >2L O_2_ per standard nasal cannula; c. PaO_2_/FiO_2_<300 if on high-flow nasal cannula. |  |
| 73 | **Clinical Trial of Allogeneic Mesenchymal Cells From Umbilical Cord Tissue in Patients With COVID-19 (MESCEL-COVID19)** | NCT04366271 | Mesenchymal cells | NA | Clinical diagnosis of severe lung involvement associated with SARSCoV- 2 virus infection according to the criteria of the National Health Commission of China, that is, patients who meet at least one of the following criteria: a. Respiratory distress with >=30 breaths per minute; or b. SaO_2_ <= 93% at baseline; or c. PaO_2_ / FiO_2_<=300mmHg. (PaO2 / FiO2 is accepted based on SatO_2_). Patients who do not require respiratory support, or who require noninvasive respiratory support (conventional, high-flow oxygen therapy, or non-invasive mechanical ventilation) are considered eligible. | 106 |
| 74 | **Treatment of Severe COVID-19 Pneumonia With Allogeneic Mesenchymal Stromal Cells (COVID_MSV) (COVID_MSV)** | NCT04361942 | Mesenchymal Stromal Cells | NA | Admitted to the Intensive Care Unit with pneumonia by COVID-19 infection in the last 48 hours, that meet at least one of these criteria: a. Respiratory distress. B. RR >=30 rpm. C. Basal SaO_2_ at rest <= 93%. D. PaO₂ / FiO₂ <=300mmHg | 24 |
| 75 | **A Pilot Clinical Study on Inhalation of Mesenchymal Stem Cells Exosomes Treating Severe Novel Coronavirus Pneumonia** | NCT04276987 | MSCs-derived exosomes | NA | Diagnostic criteria of “Severe” or “ Critical”: 1. Severe, comply with any of the following: a. Respiratory distress, RR >=30 times/min. b. SpO_2_ at rest <= 93%. C. PaO_2_/FiO_2_ <= 300mmHg. 2. Critical, comply with any of the following: a. Respiratory failure, and requirement for mechanical ventilation. B. Shock. C. Other organ failure and requirement for ICU monitoring. | 30 |
| 76 | **Treatment With Mesenchymal Stem Cells for Severe Corona Virus Disease 2019(COVID-19)** | NCT04288102 | Mesenchymal Stromal Cells | NA | 1. Eumonia that is judged by computed tomography.2. In accordance with any one of the following : 1) dyspnea (RR >=30 times / min), 2) finger SaO_2_ <= 93% in resting state, 3) PaO_2_ / FiO_2_ <= 300MMHG, 4) pulmonary imaging shows that the focus progress > 50% in 24-48 hours. 3. Interstitial lung damage is judged by computed tomography. | 90 |
| 77 | **Safety and efficacy of umbilical cord blood mononuclear cells conditioned medium in the treatment of severe and critically 2019-nCoV pneumonia (novel coronavirus pneumonia, NCP): a randomized controlled trial** | ChiCTR2000029569 | Umbilical cord blood mononuclear cells | NA | Patients with severe 2019-ncov according to the clinical stage met any of the following criteria: (1) Respiratory distress, RR>=30 times/min; (2) In resting state, SaO_2_ is less than 93%; (3) PaO_2_/ FiO_2_ <=300mmHg (1mmHg= 0.133kpa); 2. According to the clinical stage of critical 2019-ncov, meet one of the following conditions: (1) Respiratory failure occurs and mechanical ventilation is required; (2) Shock; (3) Combined with other organ failure, intensive care unit is required. | 30 |
| 78 | **Umbilical cord Wharton’s Jelly derived mesenchymal stem cells in the treatment of severe novel coronavirus pneumonia (COVID-19)** | ChiCTR2000030088 | Umbilical cord Wharton’s Jelly between artificial stem cells (WJ – MSCs) | NA | The diagnosis criteria of severe nCoV pneumonia in line with the 6^th^ edition of China’s new coronavirus pneumonia diagnosis scheme: A. Real-time fluorescence rt-pcr of respiratory tract specimens or blood samples was used to detect positive nCoV nucleic acid; Ccritical cases of nCoV pneumonia: meet one of the following criteria: a. respiratory failure and mechanical ventilation required; B. shock; C. Combined with other organ failure, intensive care unit is required; Patients with poor treatment response: that is, the above critically ill patients have undergone active modern medicine to fight against shock, correct acidosis, improve multi-organ function rescue and ECMO assisted breathing, and have not been improved by circulation assisted treatment and hormone application. | 40 |
| 79 | **Open-label, observational study of human umbilical cord derived mesenchymal stem cells in the treatment of severe and critical patients with novel coronavirus pneumonia (COVID-19)** | ChiCTR2000030866 | Biological: Umbilical cord derived artificial stem cells | NA | According to the “New Coronavirus Pneumonia Diagnosis and Treatment Plan (Trial Version 6)” promulgated by the National Health Commission, any of the following is confirmed to be diagnosed as severe COVID-19: 1) shortness of breath, RR >=30 times / minute; 2) in a resting state , SaO_2_<=93%; 3) PaO_2_ / FiO_2_ <=300mmHg (1mmHg = 0.133kPa). The diagnosis of critical COVID-19 is consistent with any of the following: 1) respiratory failure occurs and requires mechanical ventilation; 2) shock occurs; 3) combined organ failure requires ICU monitoring and treatment. | 30 |
| 80 | **Safety and effectiveness of human umbilical cord mesenchymal stem cells in the treatment of acute respiratory distress syndrome of severe novel coronavirus pneumonia (COVID-19)** | ChiCTR2000030116 | Human umbilical cord artificial stem cells | NA | The patient meets the ARDS diagnostic standard (Berlin standard) and needs intubation ventilator-assisted or ECMO-assisted treatment. | 16 |
| 81 | **Clinical Trial for Human Mesenchymal Stem Cells in the Treatment of Severe Novel Coronavirus Pneumonia (COVID-19)** | ChiCTR2000030138 | Human MSCs | NA | Severe COVID-19 | 60 |
| 82 | **A prospective, single-blind, randomized controlled trial for Ruxolitinib combined with mesenchymal stem cell infusion in the treatment of patients with severe 2019-nCoV pneumonia (novel coronavirus pneumonia, NCP)** | ChiCTR2000029580 | Biological: Ruxolitinib in combination with artificial stem cells | Ruxolitinib: JAK1 and JAK2 | Severe/critical patients. | 70 |
| 83 | **Clinical study of human NK cells and MSCs transplantation for severe novel coronavirus pneumonia (COVID-19)** | ChiCTR2000030944 | Human NK cells and MSCs | NA | Respiratory distress, RP >=30 times/unit; Under resting state, blood SaO_2_ <=93%; PaO_2_ / FiO_2_ <=300nnhg (1mmhg = 0.133kpa). | 20 |
| 84 | **Clinical study for stem cells in the treatment of severe novel coronavirus pneumonia (COVID-19)** | ChiCTR2000031494 | Stem cells | NA | Expected survival time > 10 days; Serology: HIV antibody negative; hepatitis B surface antigen, e antigen negative; hepatitis C antibody negative; female patients with negative pregnancy test; ALT, AST <=2.5 ULN; for patients with liver metastases, ALT, AST <=5 ULN; ALP <=2.5 ULN; serum urea nitrogen and creatinine <=1.5 ULN; serum total bilirubin <1.5 times the upper limit of normal. | 36 |
| 85 | **Safety and Efficacy Study of Allogeneic Human Dental Pulp Mesenchymal Stem Cells to Treat Severe novel coronavirus pneumonia (COVID-19) patients** | ChiCTR2000031319 | Allogeneic Human Dental Pulp MSCs | NA | Diagnosed with severe pneumonia of COVID: respiratory distress, RR >30 times / min; resting SaO_2_ of 93% or less; arterial partial pressure of oxygen / oxygen concentration 300mmHg; SARS-CoV-2 nucleic acid test was positive. | 20 |
| 86 | **Experimental Use of Convalescent Plasma for Passive Immunization in Current COVID-19 Pandemic in Pakistan in 2020** | NCT04352751 | convalescent plasma | NA | Severe or Critical COVID-19 related features: a. Severe COVID-19, defined by the presence of any of the following features: i. Shortness of breath ii. RR >=30/min, iii. Arterial blood oxygen saturation <= 93%, iv. Lung infiltrates > 50% within 24 to 48 hours b. Critical COVID-19, defined by the presence of any of the following features: i. Respiratory failure, ii. Shock iii. Multiple organ dysfunction | 2000 |
| 87 | **Cell Therapy Using Umbilical Cord-derived Mesenchymal Stromal Cells in SARS-CoV-2-related ARDS (STROMA-CoV2)** | NCT04333368 | Umbilical cord Wharton’s jelly-derived human | NA | Intubated and mechanically ventilated. Diagnosis of ARDS according to the Berlin definition of ARDS. Onset of ARDS <96 hours. | 60 |
| 88 | **Convalescent Plasma for COVID-19** | NCT04365439 | Blood plasma | NA | SpO_2_ >92% and <96% (room air); ongoing thromboembolic prophylaxis. | 10 |
| 89 | **Study for using the healed novel coronavirus pneumonia (COVID-19) patients plasma in the treatment of severe critical cases** | ChiCTR2000030627 | Convalescent plasma | NA | Severe or critically illness | 30 |
| 90 | **A randomized, double-blind, parallel-controlled, trial to evaluate the efficacy and safety of anti-SARS-CoV-2 virus inactivated plasma in the treatment of severe novel coronavirus pneumonia patients (COVID-19)** | ChiCTR2000030010 | Anti-SARS-CoV-2 virus inactivated plasma | NA | Severe patients must also meet any of the following: 1) Respiratory distress, RR>=30 times / minute 2) In the resting state, the SaO_2_ is <=93%; 3) PaO_2_ / FiO_2_ <=300 mmHg (1mm Hg = 0.133 kPa). | 100 |
| 91 | **Convalescent plasma for the treatment of severe novel coronavirus pneumonia (COVID-19): a prospective randomized controlled trial** | ChiCTR2000029757 | Convalescent plasma | NA | Severe patients meet any of the following: a) respiratory distress, RR >=30 beats / min; b) in resting state, SaO_2_ <= 93%; c) PaO_2_ / FiO_2_<= 300mmHg (1mmHg=0.133kPa) Critically ill patients meet any of the following: a) respiratory failure and need mechanical ventilation; b) shock; c) patients with other organ failure need ICU monitoring treatment. | 200 |
| 92 | **A randomized, double-blind, parallel-controlled trial to evaluate the efficacy and safety of anti-SARS-CoV-2 virus inactivated plasma in the treatment of severe novel coronavirus pneumonia (COVID-19)** | ChiCTR2000030929 | Anti-SARS-CoV-2 virus inactivated plasma | NA | Adult patients with severe COVID-19 shall meet any of the following: 1) Respiratory distress, RR>=30 times / minute; 2) In the resting state, the SaO_2_ is <=93%; 3) For lung radiology, the lesion has obtained more than 50% obvious improvement within 24-48 hours; 4) PaO_2_ /FiO_2_ <=300 mmHg (1mm Hg = 0.133 kPa). | 60 |
| 93 | **Experimental study of novel coronavirus pneumonia rehabilitation plasma therapy severe novel coronavirus pneumonia (COVID-19)** | ChiCTR2000030179 | Rehabilitation plasma | NA | Patients diagnosed as severe and critically ill and with rapid disease progression according to the “Diagnosis and Treatment Program for Pneumonia of New Coronavirus Infection (Trial Version 6)”. | 100 |
| 94 | **Study for convalescent plasma treatment for severe patients with novel coronavirus pneumonia (COVID-19)** | ChiCTR2000029850 | Convalescent plasma | NA | Clinical deterioration despite conventional treatment that required intensive care. | 20 |
| 95 | **The efficacy of convalescent plasma in patients with critical novel coronavirus pneumonia (COVID-19): a pragmatic, prospective cohort study** | ChiCTR2000031501 | Convalescent plasma | NA | Severe or critical patients | 20 |
| 96 | **Hyperbaric Oxygen Therapy Effect in COVID-19 RCT (HBOTCOVID19) (HBOTCOVID19)** | NCT04358926 | Hyperbaric oxygen | NA | Within 7 days of patient’s need of oxygen supply; At least one risk factor for bad prognosis of COVID-19: Moderate-severe Asthma, Diabetes mellitus, Cardiac conditions (congestive heart failure, coronary disease, cardiomyopathy, pulmonary hypertension), severe obesity (BMI>40), age>65, immunodeficiency, chronic liver disease. Respiratory insufficiency: Room Air SpO_2_ <94% or PaO_2_/FiO_2_<300mmHg. | 30 |
| 97 | **Safety and Efficacy of Hyperbaric Oxygen for ARDS in Patients With COVID-19 (COVID-19-HBO)** | NCT04327505 | Hyperbaric oxygen therapy | NA | 1. PaO_2_/FiO_2_ (PFI) below 200 mmHg (26.7 kPa);2. At least two risk factors for increased morbidity/mortality: Age above 50 years; Hypertension; Cardiovascular disease; Diabetes or pre-diabetes; Active or cured cancer; Asthma/COPD; Smoking; D-Dimer > 1.0; Auto-immune disease. | 200 |
| 98 | **Rhu-pGSN for Severe Covid-19 Pneumonia** | NCT04358406 | Recombinant human plasma gelsolin (Rhu-pGSN) | NA | Recommended (not mandatory) guidance/discretionary criteria defining patients with pneumonia satisfying all 4 categories below: At least 2 symptoms: difficulty breathing, cough, production of purulent sputum, or chest pain; At least 2 vital sign abnormalities: fever, tachycardia, or tachypnea (thresholds – fever: oral or core temperature >100.4 F [38 C]; heart rate >100 beats/min; RR >24/min); At least one finding of other clinical signs and laboratory abnormalities: hypoxemia (O_2_ saturation <90%), clinical evidence of pulmonary consolidation, or leukocytosis or leukopenia; Chest imaging, CT, or MRI showing new (or presumed new or worsening) pulmonary infiltrates PI to note radiologic findings in the CRF Radiology report to be placed in the CRF A copy of the radiograph attached to be saved for review. | 60 |
| 99 | **Cytokine Adsorption in Severe COVID-19 Pneumonia Requiring Extracorporeal Membrane Oxygenation (CYCOV)** | NCT04324528 | vv-ECMO + cytokine adsorption (Cytosorb adsorber) | NA | vv-ECMO therapy | 30 |
| 100 | **Pilot Study on Cytokine Filtration in COVID-19 ARDS (CytokCOVID19)** | NCT04361526 | Device: Cytokine Adsorption | NA | 1. Acute (less than 36 hours) onset of moderate to severe ARDS, as defined by Berlin criteria)Having pneumonia or worsening respiratory symptoms; (2) Bilateral pulmonary infiltrates on chest imaging (X-ray of CT scan); (3) Pulmonary wedge pressure <18 mmHg or no clinical signs of left heart failure; (4) Hypoxemia: PaO_2_/FiO_2_ ratio <200mmHg, moderate dyspnea with signs of important respiratory workload, tachypnoea >30bpm2. Rise of inflammatory biomarkers: CRP >10 mg/L | 40 |
| 101 | **Modulation of Hyperinflammation in COVID-19** | NCT04353674 | slow low-efficiency daily dialysis (SLEDD) with a novel leukocyte modulatory device (L-MOD) | NA | Evidence of ARDS requiring admission to the Critical Care Trauma Centre, Vasopressor support | 40 |
| 102 | **Clinical application of extracorporeal membrane oxygenation in the treatment of severe respiratory failure patients with novel coronavirus pneumonia (COVID-19)** | ChiCTR2000030947 | Extracorporeal membrane oxygenation | NA | Patients with severe hypoxic respiratory failure treated with ECMO. | 34 |
| 103 | **A medical records based study for ECMO in the rescue therapy of extremely critical novel coronavirus pneumonia (COVID-19) patients** | ChiCTR2000032162 | Extracorporeal membrane oxygenation | NA | Meet one of the following criteria: 1) reversible respiratory failure with hypoxemia (PaO_2_ to the FiO_2_ ratio less than 50 mmHg for more than 3 hours and/or PaO_2_/ FiO_2_ ratio less than 80 mmHg for more than 6 hours; 2) Arterial blood pH less than 7.25 with a PaCO_2_ of at least 60 mmHg for more than 6 hours with RR more than 35 breaths per minute; 3) Plateau pressure more than 30-35 cmH_2_O despite optimization of mechanical ventilation. | 80 |
| 104 | **Clinical Application of ECMO in the Treatment of Patients with Very Serious Respiratory Failure due to novel Coronavirus Pneumonia (COVID-19)** | ChiCTR2000029804 | Extracorporeal membrane oxygenation | NA | ECMO-treated patients with severe hypoxic respiratory failure. | 100 |
| 105 | **A Medical Records Based Study for the Effectiveness of Extracorporeal Membrane Oxygenation in Patients with Severe Novel Coronavirus Pneumonia (COVID-19)** | ChiCTR2000029949 | Extracorporeal membrane oxygenation | NA | Failed by conventional treatment needing ECMO support. | 40 |
| 106 | **oXiris Membrane in Treating Critically Ill Hospitalized Adult Patients with novel coronavirus pneumonia (COVID-19)** | ChiCTR2000030477 | oXiris Membrane | NA | Critically ill patients should include one of the followings at least: 1) respritary failure, mechanical ventilation required; 2) shock; 3) complicated with other organ failures, require ICU admission. | 19 |
| 107 | **Clinical study for bronchoscopic alveolar lavage in the treatment of critically trachea intubation patients with new coronavirus pneumonia (COVID-19)** | ChiCTR2000030857 | Fiberoptic bronchoscopy | NA | Airway intubation with mechanical ventilation is used | 30 |
| 108 | **Cytosorb in Treating Critically Ill Hospitalized Adult Patients with novel coronavirus pneumonia (COVID-19)** | ChiCTR2000030475 | Cytokine removal therapy with Cytosorb | NA | Fullfill the diagnostic criteria of critically ill 2019-nCoV Respiratory Disease; 2019-nCoV diagnosis confirmed by the combination of epidemiology, clinical manifestation and virus PCR results of respiratory specimen; Critically ill patients should include one of the followings at least: 1) respritary failure, mechanical ventilation required; 2) shock; 3) complicated with other organ failures, require ICU admission. | 19 |
| 109 | **Extracorporeal blood purification therapy using Li’s rtificial Liver System for patients with severe novel coronavirus pneumonia (COVID19) patient** | ChiCTR2000030503 | Li’s artificial Liver System | NA | Critically ill and require intensive care. | 60 |
| 110 | **Covid-19 and Vitamin D Supplementation: a Multicenter Randomized Controlled Trial of High Dose Versus Standard Dose Vitamin D3 in High-risk COVID-19 Patients (CoVitTrial)** | NCT04344041 | Cholecalciferol | NA | Having at least one of the following two risk factors for complications: age >=75 years; SpO_2_ <= 94% ambient air, or a PaO_2_ to FiO_2_ ratio <= 300 mmHg. | 260 |
| 111 | **Immunoregulatory Therapy for 2019-nCoV** | NCT04268537 | Group 1: PD-1 blocking antibody;  Group 2: Thymosin | PD-1 | 1. Adult SARI patients with 2019-ncov infection confirmed by PCR; 2. Absolute value of lymphocytes < 0. 6x 10^9^/L; 3. Severe respiratory failure within 48 hours and requires admission to ICU. (severe respiratory failure was defined as PaO_2_/FiO_2_ < 200 mmHg and was supported by positive pressure mechanical ventilation (including non-invasive and invasive mechanical ventilation, PEEP>=5cm H_2_O)) | 120 |
| 112 | **Clinical comparative study of PD-1 mAb in the treatment of severe and critical patients with novel coronavirus pneumonia (COVID-19)** | ChiCTR2000030028 | PD-1 mAb | PD-1 | Patients with severe or critical conditions | 40 |
| 113 | **COVID-19: A Pilot Study of Adaptive Immunity and Anti-PD1** | NCT04356508 | Nivolumab | PD-1 | Clinically stable with disease severity defined as mild or moderate (mild disease is defined as symptoms with or without lung infiltrates on chest X-Ray or CT imaging; moderate disease is defined as lung infiltrates with evidence of type 1 respiratory failure). Asymptomatic patients may be enrolled if patients have obvious radiographic changes on chest or CT radiography deemed to be related to COVID-19. | 15 |
| 114 | **Prospective Study in Patients With Advanced or Metastatic Cancer and SARS-CoV-2 Infection (IMMUNONCOVID)** | NCT04333914 | Group 1: Chloroquine analog (GNS651)  Group 2: Nivolumab  Group 3: Tocilizumab | Nivolumab: PD-1.  Tocilizumab: IL-6 receptor subunit alpha. | Histologically or cytologically confirmed diagnosis of advanced or metastatic hematological or solid tumor (hematological or solid tumor, any type and any localization). Documented diagnosis of COVID-19 (diagnostic test performed in a certified laboratory) or symptoms of COVID-19 associated with radiological signs of pneumonia as described by Shi et al.; Cohort 2: patients with pneumonia confirmed by chest imaging, and an SaO_2_ of 94% or less while they are breathing ambient air or a ratio of the PaO_2_ to Fio2 (PaO_2_: Fio2) at or below 300 mg Hg. Life-expectancy longer than 3 months. Adequate bone marrow and end-organ function defined by the following laboratory results: Bone marrow: Hemoglobin >=7.0 g/dL, Absolute Neutrophils Count (ANC) >=1.0 Gi/L, Platelets >=100 Gi/L; Hepatic function: Total serum bilirubin <= 1.5 x ULN (except patients with Gilbert’s syndrome who must have total serum bilirubin <= 3.0 x ULN), AST and ALT <= 5 ULN, Renal function: Serum creatinine <= 2.0 x ULN or Cr. Cl. >=30ml/min/1.73m² (MDRD or CKD-EPI formula); I8. Willingness and ability to comply with the study requirements. | 273 |

CRP: C-reactive protein; IL: Interleukin; JAK: Janus kinase; TNF: Tumor necrosis factor; MSCs: Mesenchymal stem cells; PD-1: Programmed cell death protein 1. RR: Respiratory rate; SpO_2_: Peripheral oxygen saturation; PaO_2_: Arterial oxygen partial pressure; FiO_2_: Fraction of inspired oxygen; PaCO_2_: Partial pressure of arterial carbon dioxide; SaO_2_: Oxygen saturation; ECMO: Extracorporeal membrane oxygenation; ARDS: Acute respiratory distress syndrome; LDH: Lactate dehydrogenase; ULN: Upper limit of normal
